# Oncogene-induced TIM-3 ligand expression dictates susceptibility to anti–TIM-3 therapy in mice

**DOI:** 10.1172/JCI177460

**Published:** 2024-06-25

**Authors:** Nana Talvard-Balland, Lukas M. Braun, Karen O. Dixon, Melissa Zwick, Helena Engel, Alina Hartmann, Sandra Duquesne, Livius Penter, Geoffroy Andrieux, Lukas Rindlisbacher, Andrea Acerbis, Jule Ehmann, Christoph Köllerer, Michela Ansuinelli, Andres Rettig, Kevin Moschallski, Petya Apostolova, Tilman Brummer, Anna L. Illert, Markus A. Schramm, Yurong Cheng, Anna Köttgen, Justus Duyster, Hans D. Menssen, Jerome Ritz, Bruce R. Blazar, Melanie Boerries, Annette Schmitt-Gräff, Nurefsan Sariipek, Peter Van Galen, Joerg M. Buescher, Nina Cabezas-Wallscheid, Heike L. Pahl, Erika L. Pearce, Robert J. Soiffer, Catherine J. Wu, Luca Vago, Burkhard Becher, Natalie Köhler, Tobias Wertheimer, Vijay K. Kuchroo, Robert Zeiser

**Affiliations:** 1 Department of Internal Medicine I, Faculty of Medicine and Medical Center,; 2 CIBSS–Centre for Integrative Biological Signalling Studies, and; 3 Faculty of Biology, University of Freiburg, Freiburg, Germany.; 4 Gene Lay Institute of Immunology and Inflammation, Brigham and Women’s Hospital, Massachusetts General Hospital, and Harvard Medical School, Boston, Massachusetts, USA.; 5 Department of Biomedicine, University of Basel and University Hospital of Basel, Basel, Switzerland.; 6 Department of Medical Oncology, Dana-Farber Cancer Institute, and Harvard Medical School, Boston, Massachusetts, USA.; 7 Department of Hematology, Oncology, and Tumorimmunology, Campus Virchow Klinikum, Berlin, Charité–Universitätsmedizin Berlin, Corporate member of Freie Universität Berlin and Humboldt-Universität zu Berlin, Berlin, Germany.; 8 Institute of Medical Bioinformatics and Systems Medicine, Medical Center, University of Freiburg, Faculty of Medicine, University of Freiburg, Freiburg, Germany.; 9 Institute of Experimental Immunology, University of Zurich, Zurich, Switzerland.; 10 Hematology, Department of Translational and Precision Medicine, Sapienza University of Rome, Rome, Italy; 11 German Cancer Consortium (DKTK) Partner Site Freiburg, a partnership between German Cancer Research Center (DKFZ) and Medical Center, University of Freiburg, Freiburg, Germany.; 12 The Bloomberg-Kimmel Institute for Cancer Immunotherapy at Johns Hopkins University School of Medicine, Baltimore, Maryland, USA.; 13 Signalling Research Centres BIOSS and CIBSS–Centre for Integrative Biological Signalling Studies, University of Freiburg, Freiburg, Germany.; 14 Institute of Molecular Medicine and Cell Research (IMMZ), Freiburg, Germany.; 15 Department of Internal Medicine III, Klinikum Rechts der Isar, Technical University of Munich, Munich, Germany.; 16 Department of Rheumatology and Clinical Immunology, Medical Center,; 17 Institute of Genetic Epidemiology, Faculty of Medicine and Medical Center–University of Freiburg, Freiburg, Germany.; 18 Novartis Pharma, Basel, Switzerland.; 19 University of Minnesota, Department of Pediatrics, Division of Blood and Marrow Transplant & Cellular Therapy, Minneapolis, Minnesota, USA.; 20 University of Freiburg, Freiburg, Germany.; 21 Division of Hematology, Brigham and Women’s Hospital, Boston, Massachusetts, USA.; 22 Max-Planck Institute of Immunobiology and Epigenetics, Freiburg, Germany.; 23 Unit of Immunogenetics, Leukemia Genomics and Immunobiology, Division of Immunology, Transplantation and Infectious Disease, IRCCS San Raffaele Scientific Institute, Milano, Italy.

**Keywords:** Transplantation, Bone marrow transplantation

## Abstract

Leukemia relapse is a major cause of death after allogeneic hematopoietic cell transplantation (allo-HCT). We tested the potential of targeting T cell (Tc) immunoglobulin and mucin-containing molecule 3 (TIM-3) for improving graft-versus-leukemia (GVL) effects. We observed differential expression of TIM-3 ligands when hematopoietic stem cells overexpressed certain oncogenic-driver mutations. Anti–TIM-3 Ab treatment improved survival of mice bearing leukemia with oncogene-induced TIM-3 ligand expression. Conversely, leukemia cells with low ligand expression were anti–TIM-3 treatment resistant. In vitro, TIM-3 blockade or genetic deletion in CD8^+^ Tc enhanced Tc activation, proliferation, and IFN-γ production while enhancing GVL effects, preventing Tc exhaustion, and improving Tc cytotoxicity and glycolysis in vivo. Conversely, TIM-3 deletion in myeloid cells did not affect allogeneic Tc proliferation and activation in vitro, suggesting that anti–TIM-3 treatment–mediated GVL effects are Tc induced. In contrast to anti–programmed cell death protein 1 (anti–PD-1) and anti–cytotoxic T lymphocyte–associated protein 4 (anti–CTLA-4) treatment, anti–TIM-3-treatment did not enhance acute graft-versus-host disease (aGVHD). TIM-3 and its ligands were frequently expressed in acute myeloid leukemia (AML) cells of patients with post–allo-HCT relapse. We decipher the connections between oncogenic mutations found in AML and TIM-3 ligand expression and identify anti–TIM-3 treatment as a strategy for enhancing GVL effects via metabolic and transcriptional Tc reprogramming without exacerbation of aGVHD. Our findings support clinical testing of anti–TIM-3 Ab in patients with AML relapse after allo-HCT.

## Abstract 2

Specific acute myeloid leukemia oncogenic mutations that induce TIM-3 ligands confer susceptibility to anti–TIM-3 therapy, an intervention that reverses the T cell phenotype and metabolism.

## Graphical abstract

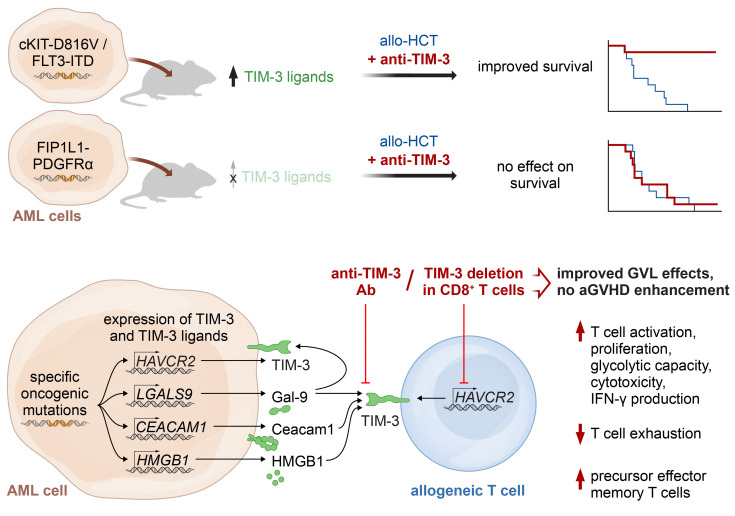

## Introduction

Acute myeloid leukemia (AML) relapse is the main cause of death after allogeneic hematopoietic cell transplantation (allo-HCT) (1). Mechanisms promoting relapse include MHC class II downregulation (2, 3), loss of mismatched HLA (4), immune checkpoint ligand upregulation (3), reduced IL-15 production (5), and leukemia-derived lactic acid release (6) among others (reviewed in ref. 7). A longstanding approach to addressing relapse is treatment with cellular therapy, such as donor lymphocyte infusions (DLI), either alone or with hypomethylating agents (HMA) (8, 9). A retrospective analysis on response to DLI reported complete response (CR) rates of 17% for patients with AML (10), and another large retrospective, multicenter study reported a 2-year overall survival (OS) upon DLI treatment of 20% versus 9% upon chemotherapy-only treatment (9). These reports indicate that donor T cells (Tc) can exert graft-versus-leukemia (GVL) effects, but also demonstrate the high unmet medical need for therapies that enhance GVL effects. Current pharmacological approaches for prevention and treatment of AML relapse include the use of FMS-like tyrosine kinase 3 (FLT3) kinase inhibitors (5, 11, 12), immune checkpoint inhibitors (ICIs) (13–17), HMA (8, 18), B cell lymphoma 2 (BCL-2) inhibitors (19), mouse double minute 2 (MDM2) inhibition (20), and others (21).

T cell immunoglobulin and mucin-containing molecule 3 (TIM-3) has recently emerged as an inhibitory receptor (22, 23) and, more broadly, as a marker for Tc dysfunction in cancer (24). It was recently described that TIM-3 may also play an important role in regulating immune responses in solid tumors, as evidenced by recent data from mouse models of melanoma (23).

In addition to its role in immune cells, TIM-3 is a promising target in AML because it is highly expressed on leukemic stem cells (LSCs) (25). Functionally, TIM-3 and its ligand, galectin-9 (Gal-9), induce an autocrine loop that is essential for LSC self-renewal and AML development (26). Additionally, refractoriness to treatment correlated with TIM-3 expression in AML (27). Early clinical trials testing TIM-3 blockade using the humanized IgG4 anti–TIM-3 Ab (sabatolimab, MBG453, Novartis) in combination with decitabine in 60 evaluable patients with AML outside of the allo-HCT setting reported CR rates of 35.3% (28). The contribution of TIM-3 in LSCs contrary to Tc for clinical activity of anti–TIM-3 Ab remains unclear.

Using an anti–TIM-3 blocking Ab, we observed that TIM-3 inhibition enhanced the survival of leukemia-bearing mice after allo-HCT and reduced exhausted TIM-3^+^PD-1^+^ (where PD-1 indicates programmed cell death protein 1) Tc frequencies. Importantly, we report that cell-specific TIM-3 deletion in CD8^+^ Tc (*Havcr2^fl/fl^;E8i^cre/+^*) caused enhanced Tc proliferation, activation, effector phenotype, and antileukemia immunity.

## Results

### Differential TIM-3 ligand expression in hematopoietic stem cells upon oncogene activation confers susceptibility to anti–TIM-3 Ab therapy in multiple AML models.

**Figure 1:**
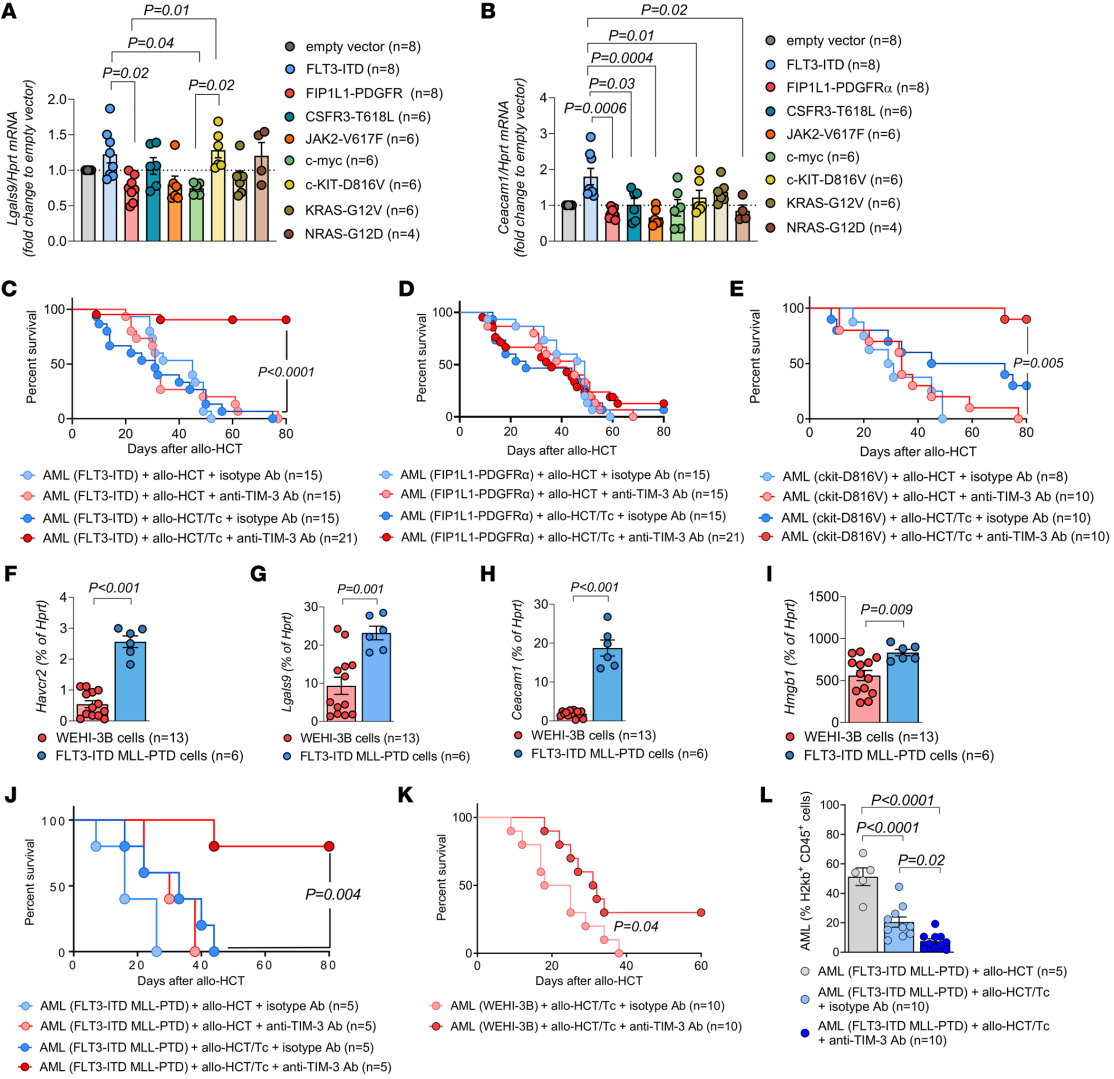
TIM-3 inhibition enhances GVL effects in different AML models. Murine primary HSCs (C57BL/6) were transduced with the indicated oncogenes or gene fusions. RNA expression of (**A**) *Lgals9* and (**B**) *Ceacam1* was determined by quantitative PCR (qPCR). Gene expression was normalized to *Hprt*. Fold change was calculated in comparison with empty vector (dotted line). Results are represented as mean ± SEM from 3 independent experiments. *P* values were calculated using Kruskal-Wallis 1-way ANOVA multiple-comparisons test. (**C**–**E**) Kaplan-Meier plots showing mouse survival in the indicated groups. BALB/c recipient mice were injected i.v. with 7 × 10^4^ oncogene-transduced HSCs and 5 × 10^6^ allogeneic BM and/or Tc when indicated. All mice were treated i.p. with isotype Ab or anti–TIM-3 Ab as indicated. *P* values were calculated using 2-sided Mantel-Cox test. (**F**–**I**) RNA expression of (**F**) *Havcr2*, (**G**) *Lgals9*, (**H**) *Ceacam1*, and (**I**) *Hmgb1* in WEHI-3B (*n* = 13) and in FLT3-ITD MLL-PTD cells (*n* = 6) was analyzed by qPCR. Gene expression is shown as percentage of *Hprt*. *P* values were calculated using an unpaired Student’s *t* test. (**J** and **K**) Kaplan-Meier plot showing mouse survival in the indicated groups. Recipient mice were injected i.v. with FLT3-ITD MLL-PTD (**J**) or WEHI-3B cells (**K**) and 5 × 10^6^ allogeneic BM and/or Tc and treated with either isotype Ab or anti–TIM-3 Ab, as indicated. *P* value was calculated using 2-sided Mantel-Cox test. (**K**) Kaplan-Meier plots showing mouse survival in the indicated groups. BALB/c recipient mice were injected with WEHI-3B cells and 5 × 10^6^ allogeneic BM and Tc and treated with isotype Ab (*n* = 10) or anti–TIM-3 Ab (*n* = 10). Data are represented as 2 independent experiments. *P* value was calculated using 2-sided Mantel-Cox test. (**L**) Data are represented as mean ± SEM of CD45^+^H-2Kb^+^ cell frequency in the BM at day 23 after allo-HCT. C57BL/6 recipient mice were injected with FLT3-ITD MLL-PTD cells and allogeneic BM and/or Tc and treated with isotype Ab (*n* = 10) or anti–TIM-3 Ab (*n* = 10). *P* values were calculated using 1-way ANOVA followed by Tukey’s multiple-comparisons test.

We previously reported that the JAK2-V617F oncogenic mutation enhances the expression of the immunosuppressive programmed death-ligand 1 (PD-L1) (29) and sought to investigate connections between oncogenes found in AML and the expression of TIM-3 (encoded by the hepatitis A virus cellular receptor 2 [*Havcr2*] gene) and its ligands. We observed that the TIM-3 ligands Gal-9 (encoded by *Lgals9*) and carcinoembryonic antigen–related cell adhesion molecule 1 (*Ceacam1*) were differentially expressed when compared with empty vector control depending on the oncogene that was introduced into hematopoietic stem cells (HSCs) of BALB/c mice, with FLT3-ITD showing the greatest upregulation of both ligands. c-KIT-D816V oncogenic-driver mutation led to a significant increase of *Lgals9* in HSCs (Figure 1, A and B). Conversely, we found no association between oncogenes and TIM-3 or TIM-3 ligand high-mobility group box 1 (*Hmgb1*) expression (Supplemental Figure 1, A and B; supplemental material available online with this article; https://doi.org/10.1172/JCI177460DS1). Consistent with the findings in primary HSCs, the transduction of the myeloblast-like murine cell line 32D with FLT3-ITD caused enhanced secretion of Gal-9, while less Gal-9 was secreted upon overexpression of FIP1L1-PDGFRα (Supplemental Figure 1C). We then transferred HSCs previously transduced with different oncogenes in mice undergoing allo-HCT and allogeneic Tc transfer and compared the efficacy of anti–TIM-3 treatment after FLT3-ITD or FIP1L1-PDGFRα Tg HSC injection. Mice were treated i.p. every 3 days from day 7 to day 28 with 150 μg of anti–TIM-3 or isotype Abs. Anti–TIM-3 treatment was efficient in mice undergoing allo-HCT carrying FLT3-ITD Tg HSCs or c-KIT-D816V Tg HSCs, but not in mice carrying FIP1L1-PDGFRα Tg HSCs (Figure 1, C–E). We observed a higher expression of TIM-3 ligands in FLT3-ITD and c-KIT-D816V Tg HSCs compared with FIP1L1-PDGFRα Tg HSCs, which is in agreement with the concept that anti–TIM-3 therapy sensitivity is associated with the expression of TIM-3 ligands.

TIM-3, Gal-9, and CEACAM1 expression levels were increased on CD8^+^ Tc at days 17 and 24 in mice injected with FLT3-ITD MLL-PTD AML cells and undergoing allo-HCT with allogeneic Tc transfer compared with untreated control mice (Supplemental Figure 1D). This increase likely reflects the activation of alloreactive Tc. Conversely, gene expression levels of *Havcr2* and *Ceacam1* did not differ on leukemia cells at different time points after allo-HCT as compared with untreated controls. *Lgals9* gene expression was only increased on day 10 after allo-HCT (Supplemental Figure 1E).

We further tested the effect of anti–TIM-3 treatment on the GVL effect in additional models using WEHI-3B AML and the previously described FLT3-ITD MLL-PTD–driven AML model (5). In all models, donor Tc are infused 2 days after allo-HCT to reproduce clinical conditions where DLI is administered during low leukemia burden, as shown previously (5, 20). FLT3-ITD MLL-PTD–driven AML cells exhibited higher TIM-3 and ligand expression compared with WEHI-3B AML cells (Figure 1, F–I). Anti–TIM-3 treatment improved the survival in both models, with a more prominent effect observed in FLT3-ITD MLL-PTD–driven AML, consistent with previous observations (Figure 1, J and K). Notably, mice treated with anti–TIM-3 Ab without allogeneic Tc transfer were not protected from FLT3-ITD MLL-PTD–driven AML (Figure 1J), indicating that anti–TIM-3 Ab requires allo-HCT to exert its effects. Anti–TIM-3 treatment also reduced the leukemia burden in FLT3-ITD MLL-PTD–driven AML, as evidenced by decreased AML cell frequency in the BM at day 23 after allo-HCT (Figure 1L). Additionally, mice treated with anti–TIM-3 Ab in the absence of allo-HCT were not protected from FLT3-ITD MLL-PTD–driven AML (Supplemental Figure 2A), supporting the hypothesis that anti–TIM-3 Ab have no direct cytotoxic effect against AML cells, but require allo-HCT. These results highlight that oncogene-driven expression of TIM-3 and its ligands contributes to disease progression and targeting TIM-3 in the presence of donor allogeneic Tc enhances the GVL effect.

### Anti–TIM-3 Ab treatment after allo-HCT reduces Tc exhaustion, increases glycolytic capacity of Tc, and enhances immune responses.

**Figure 2:**
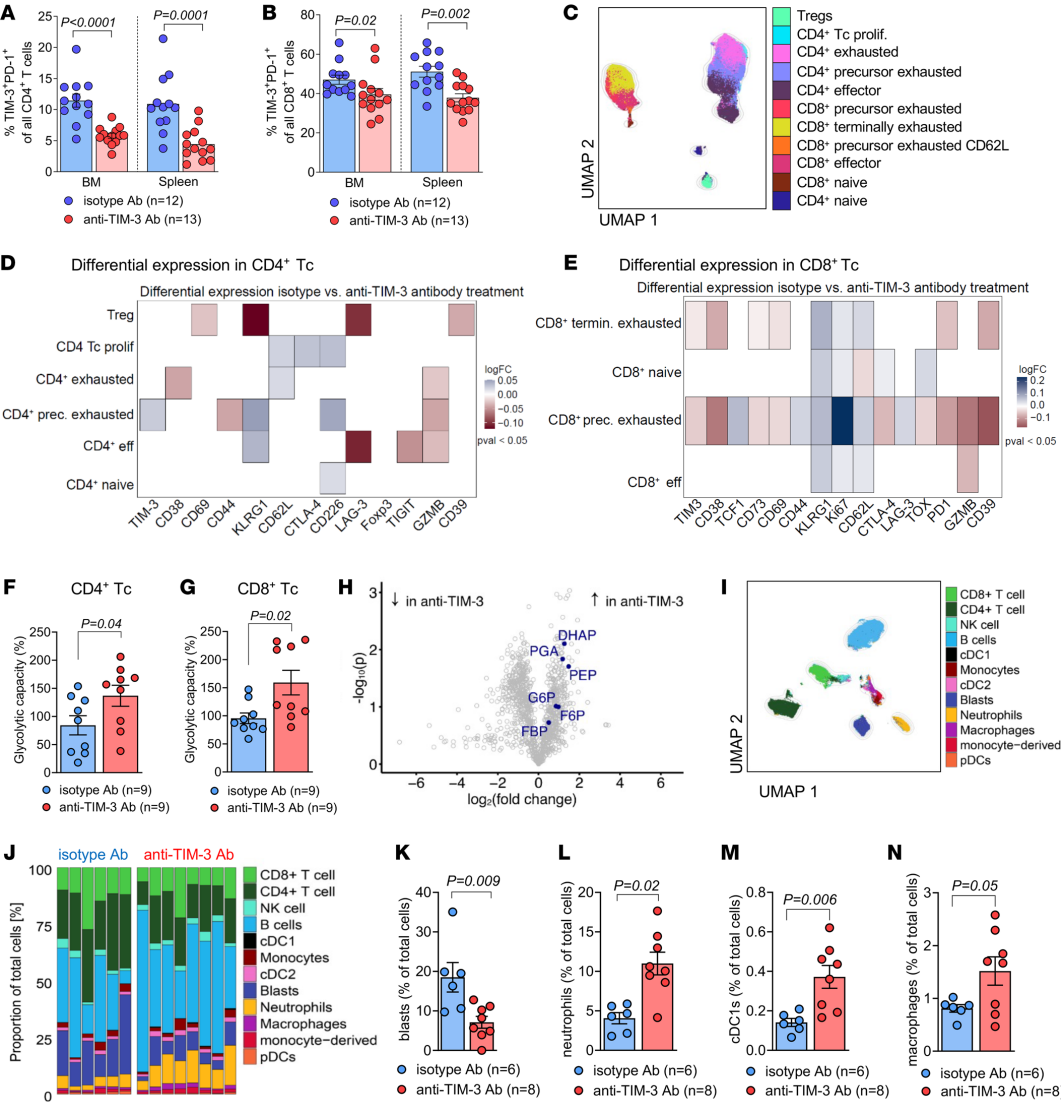
Anti–TIM-3 Ab treatment after allo-HCT reduces Tc exhaustion, increases glycolytic capacity of Tc, and induces changes in myeloid subsets. (**A** and **B**) BALB/c recipient mice were injected with WEHI-3B cells, 5 × 10^6^ allogeneic BM, and Tc and treated with isotype (*n* = 12) or anti–TIM-3 Ab (*n* = 13). The proportion of TIM-3^+^PD-1^+^ cells within CD4^+^ (**A**) or CD8^+^ Tc (**B**) in the indicated organ was determined by FC at day 23. Data are represented as mean ± SEM, and *P* values were calculated using Mann*-*Whitney *U* test. (**C**–**N**) C57BL/6 recipient mice were injected with FLT3-ITD MLL-PTD cells, 5 × 10^6^ allogeneic BM, and Tc and treated with isotype Ab or anti–TIM-3 Ab. Donor Tc were isolated from the spleen at day 23 after allo-HCT. (**C**) Analysis using high-resolution spectral FC allows UMAP visualization of the immune landscape. (**D** and **E**) Scaled expression of 25 phenotypic or functional markers using the FlowSOM algorithm among 6 CD4^+^ or 4 CD8^+^ Tc subsets. Log fold change of isotype Ab (*n* = 6) compared with anti–TIM-3 Ab (*n* = 8) treatment is shown (blue color depicts higher expression in isotype, red color higher expression in anti–TIM-3). Differentially expressed proteins with *P* < 0.05 tested with moderated *t* test of limma are presented. (**F** and **G**) Glycolytic capacity of Tc subsets at day 23 assessed by FC-based single-cell metabolism. Data are represented as mean ± SEM of *n* = 9 biological replicates for each condition. *P* values were calculated using Mann*-*Whitney *U* test. (**H**) Volcano plot of 1,249 metabolic features from nontargeted LC-MS analysis. Features that were identified as members of the KEGG module “glycolysis” are highlighted. Results show *n* = 5 isotype and *n* = 8 anti–TIM-3-treated mice. DHAP, dihydroxyacetonphosphate; PGA, phosphoglyceric acid; PEP, phosphoenolpyruvate; G6P, glucose-6-phosphate; F6P, fructose-6-phosphate; FBP, fructose-bisphosphate. (**I**) Analysis using high-resolution spectral FC allows UMAP visualization of immune cells. (**J**) Proportion of each cell subset among total cells in the respective condition. Proportion of leukemia blasts (**K**), neutrophils (**L**), cDC1s (**M**), and macrophages (**N**) among total cells (%). *P* values were calculated using unpaired Student’s *t* test.

Since Ab-dependent cell-mediated cytotoxicity (ADCC) of the anti–TIM-3 Ab on AML cells was unlikely based on the lack of efficacy against AML cells in vivo in the absence of Tc (Figure 1J), we investigated the effect of anti–TIM-3 Ab treatment on Tc phenotype in both WEHI-3B and FLT3-ITD MLL-PTD AML models. Tc exhaustion, characterized by the coexpression of PD-1 and TIM-3, has been observed in AML patients relapsing after allo-HCT (30). Using WEHI-3B cells with allo-HCT and Tc transfer followed by anti–TIM-3 or isotype Ab treatment, we found a lower frequency of exhausted TIM-3^+^PD-1^+^ donor Tc in CD4^+^ and CD8^+^ Tc subsets, in the spleen and in the BM isolated on day 23 after allo-HCT, upon anti–TIM-3 treatment compared with isotype Ab treatment (Figure 2, A and B, and Supplemental Figure 3, A–C). Importantly, this reduction in TIM-3^+^ Tc was not due to steric hindrance by the anti–TIM-3 blocking Ab, as the Ab used for flow cytometry (FC) detection recognize different epitopes, and TIM-3 staining was unaffected by anti–TIM-3 Ab treatment (Supplemental Figure 3, D and E). To extend our analysis of Tc exhaustion, we conducted high-resolution spectral FC-based analysis on Tc isolated from spleens at day 23 after allo-HCT in FLT3-ITD MLL-PTD AML–bearing mice. The analysis revealed several CD4^+^ and CD8^+^ Tc subsets (Figure 2C and Supplemental Figure 5A). By investigating both phenotypic and functional markers, we observed that anti–TIM-3 Ab treatment profoundly remodeled both CD4^+^ and CD8^+^ Tc subsets when compared with isotype Ab treatment. Notably, we found an increased expression of granzyme B in the CD8^+^ effector Tc (Teff) subset upon treatment with anti–TIM-3 Ab as well as an increased expression of CD38 and the ectonucleoside triphosphate diphosphohydrolase-1 (CD39) in precursor exhausted CD8^+^ Tc (Figure 2, D and E), which have been described as tumor-specific CD8^+^ Tc that exhibit potent antitumor activity against different solid cancers (31–34). Following our previous work showing that reduced glycolytic activity of Tc is associated with impaired GVL effects (6), we next utilized a single-cell FC-based assay to measure the metabolic response in donor Tc by quantifying protein-synthesis levels across different immune cells (35). Tc were isolated from spleens of FLT3-ITD MLL-PTD AML–bearing mice after allo-HCT and Tc transfer because of the improved survival of anti–TIM-3 Ab–treated mice in this model (Figure 1J). We observed a higher glycolytic capacity of CD4^+^ and CD8^+^ Tc upon anti–TIM-3 Ab treatment compared with isotype Ab treatment (Figure 2, F and G). Additionally, nontargeted metabolomics analysis using liquid chromatography–mass spectrometry (LC-MS) revealed elevated levels of all identified members of the Kyoto Encyclopedia of Genes and Genomes (KEGG) module “glycolysis” in Tc from anti–TIM-3 Ab–treated mice (Figure 2H). The analysis of the 10 most significantly enriched pathways revealed an enrichment of the pathway “glycolysis and gluconeogenesis” in Tc isolated from anti–TIM-3 Ab compared with isotype-treated mice (Supplemental Figure 4, A and B). Taken together, these findings indicate that anti–TIM-3 treatment enhances glycolytic activity in Tc following allo-HCT in leukemia-bearing mice.

Using high-dimensional spectral FC, we next examined the impact of anti–TIM-3 Ab treatment on the myeloid lineage phenotype in FLT3-ITD MLL-PTD AML–bearing mice that had undergone allo-HCT and Tc transfer on day 2 after allo-HCT (Figure 2I). Analysis of the spleen at day 23 after allo-HCT revealed a decreased frequency of leukemic blasts, in line with a more potent GVL effect, and a higher frequency of neutrophils upon anti–TIM-3 treatment (Figure 2, J–L). Consistent with a potential role for neutrophils in ICI-mediated GVL effects, recent evidence has suggested that neutrophils contribute to ICI-induced antitumor immune responses (36, 37). Additionally, we found an increased frequency of type-1 conventional DCs (cDC1s) and macrophages upon anti–TIM-3 compared with the isotype Ab treatment (Figure 2, M and N). cDC1s have been described as providing an essential niche to promote memory precursor Tc maintenance and differentiation in the context of anti–PDL-1 therapy (38). Furthermore, we found that the expression of TIM-3 and Gal-9 was significantly enriched in distinct myeloid cell types upon anti–TIM-3 treatment. Notably, expression of TIM-3 was increased in cDC1s and macrophages, while monocytes and macrophages were enriched for Gal-9 (Supplemental Figure 5B).

**Figure 3:**
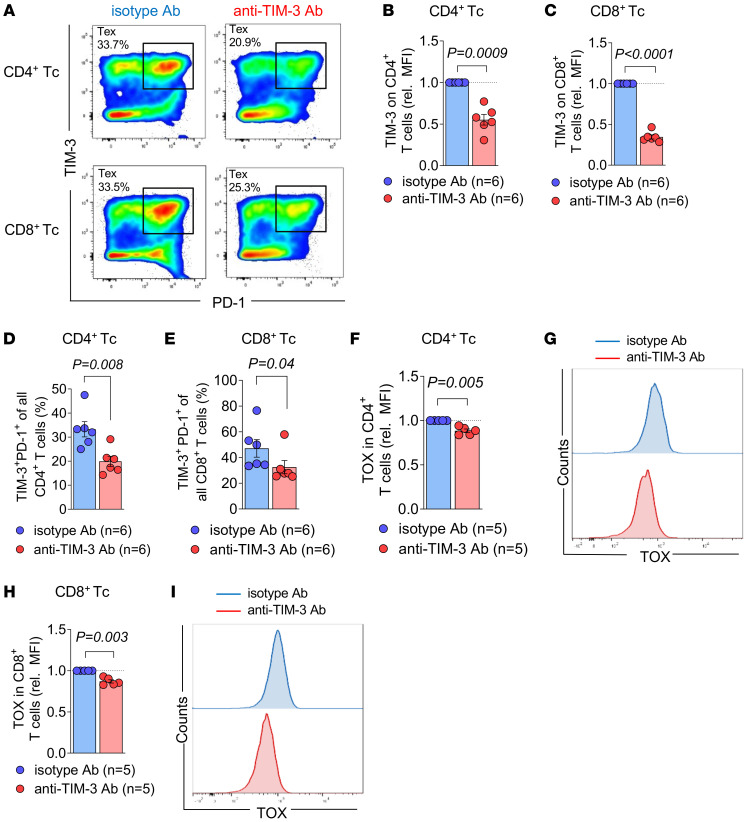
Anti–TIM-3 Ab treatment reduces Tc exhaustion in vitro. (**A**–**G**) Tc isolated from spleens (C57BL/6 mice) were continuously exposed to TCR stimulation (αCD3/CD28 beads) in vitro for 14 days in RPMI medium supplemented with 10% FBS, 1% penicillin/streptomycin, and 30 U/mL of mIL-2 to obtain highly activated/exhausted Tc. Cells were treated every second day with 10 μg/mL of isotype or anti–TIM-3 Ab. (**A**) Representative plots showing the proportion of TIM-3^+^PD-1^+^ cells among viable CD4^+^ and CD8^+^ Tc treated with isotype or anti–TIM-3, determined by FC. Relative TIM-3 protein expression in CD4^+^ Tc (**B**) and in CD8^+^ Tc (**C**) based on MFI for *n* = 6 replicates for each condition. *P* values were calculated using Wilcoxon’s signed-rank test. (**D** and **E**) Proportion (%) of TIM-3^+^PD-1^+^ within all CD4^+^ Tc (**D**) and within all CD8^+^ Tc (**E**). Data are represented as mean ± SEM for isotype Ab (*n* = 6) and anti–TIM-3 Ab (*n* = 6). *P* values were calculated using Mann*-*Whitney *U* test. (**F**) Relative TOX protein expression as relative MFI determined by FC in CD4^+^ Tc and (**G**) representative FC staining of TOX expression in CD4^+^ Tc for both isotype Ab (blue line) and anti–TIM-3 Ab treatment (red line). (**H**) Relative TOX protein expression as relative MFI determined by FC in CD8^+^ Tc and (**I**) representative staining of TOX expression in CD8^+^ Tc for both isotype Ab (blue line) and anti–TIM-3 Ab treatment (red line). Data are represented as mean ± SEM of *n* = 5 isotype treatment or *n* = 5 anti–TIM-3 treatment. *P* values were calculated using Wilcoxon’s signed-rank test.

Consistent with the effects of anti–TIM-3 treatment observed in vivo, we observed similar effects following in vitro anti-CD3/CD28–mediated Tc activation. When the isotype Ab was added to the culture, more than 30% of CD4^+^ and CD8^+^ Tc subsets displayed a phenotype of exhaustion (based on the coexpression of PD-1 and TIM-3 on Tc) after 14 days of culture (Figure 3A). Consistent with our in vivo studies, addition of anti–TIM-3 Ab in vitro resulted in reduced frequencies of exhausted Tc in both CD4^+^ and CD8^+^ subsets (Figure 3, A–E). Additionally, we observed a decreased expression of the exhaustion-associated transcription factor thymocyte selection–associated HMG BOX (TOX) in CD4^+^ and CD8^+^ Tc when the anti–TIM-3 Ab was added to the culture (Figure 3, F–I).

These findings indicate that anti–TIM-3 Ab treatment induces several changes in both Tc and myeloid cell populations. We found phenotypic features of Tc exhaustion to be reduced in vitro and in vivo. Accordingly, we observed increased effector functions in different Tc subsets in vivo, which is in line with a more effective elimination of AML blasts.

### Genetic TIM-3 deletion in CD8^+^ Tc enhances Tc activation, proliferation, and IFN-γ production as well as GVL effects.

**Figure 4:**
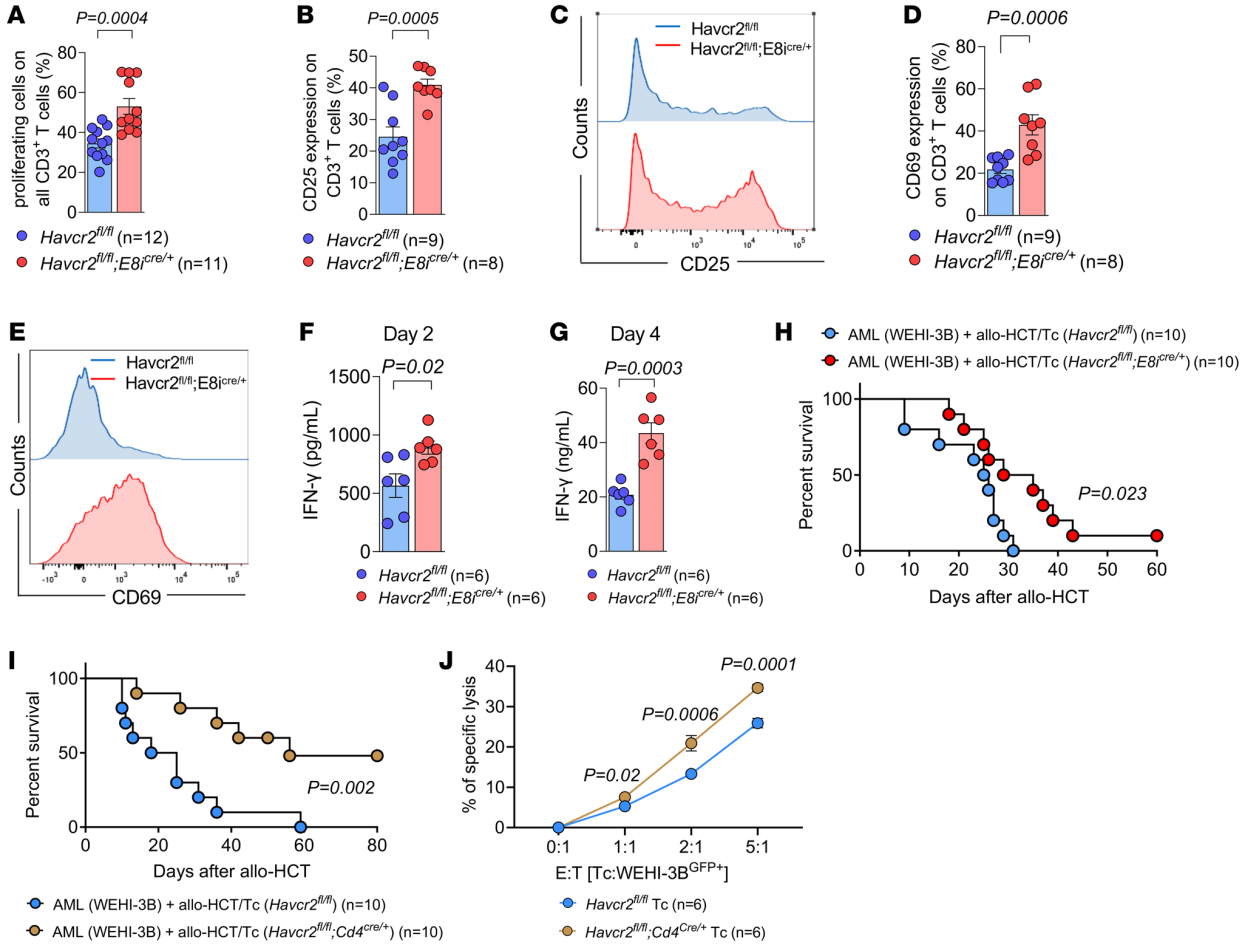
Genetic TIM-3 deletion in CD8^+^ Tc enhances Tc activation, proliferation, and IFN-γ production as well as GVL effects. (**A**–**E**) Tc labeled with CTV (responders, C57BL/6) were cocultured for 6 days with allogeneic non-CD3 cells (stimulators, BALB/c). Responder cells were isolated from mice carrying TIM-3 deletion in CD8^+^ Tc (*Havcr2^fl/fl^;E8i^cre/+^*) as described (23). (**A**) Proportion of proliferative cells was quantified at day 6 as the percentage of CTV^lo^ cells in *Havcr2^fl/fl^;E8i^cre/+^* CD3^+^ responder Tc. (**B**) CD25 expression in proliferative CD3^+^ responder cells was quantified at day 6 by FC. (**C**) Representative staining of CD25 expression in *Havcr2^fl/fl^* (blue line) and in *Havcr2^fl/fl^;E8i^cre/+^* cells (red line). (**D**) CD69 expression in proliferative CD3^+^ responder cells was quantified at day 6 by FC. (**E**) Representative staining of CD69 expression in *Havcr2^fl/fl^* (blue line) and in *Havcr2^fl/fl^;E8i^cre/+^* cells (red line). (**F** and **G**) Production of IFN-γ in the supernatant of the culture analyzed at the indicated time points by ELISA. *P* values were calculated using an unpaired Student’s *t* test. (**H** and **I**) Kaplan-Meier plots showing survival of mice in the indicated groups. BALB/c recipient mice were injected i.v. with WEHI-3B AML cells (BALB/c background) and (**H**) allogeneic *Havcr2^fl/fl^* (*n* = 10) or *Havcr2^fl/fl^;E8i^cre/+^* (*n* = 10) BM and Tc or (**I**) allogeneic *Havcr2^fl/fl^* (*n* = 10) or *Havcr2^fl/fl^;Cd4^cre/+^* (*n* = 10) BM and Tc. Data were pooled from 2 independent experiments, and *P* values were calculated using the 2-sided Mantel-Cox test. (**J**) Percentage of specific lysis of αCD3/CD28-activated Tc isolated from Havcr2^cKO^ mice in contact with WEHI-3B cells. E:T (effector [Tc] to target [WEHI-3B cell]) ratio was titrated between 5:1 and 1:1, as indicated. Individual values are shown and mean ± SD of *Havcr2^fl/fl^* (*n* = 6) or *Havcr2^fl/fl^;Cd4^cre/+^* (*n* = 6). *P* values were calculated using 2-way ANOVA followed by Šidák’s multiple-comparisons test.

Since the anti–TIM-3 Ab will block TIM-3 on multiple cell types, we next used TIM-3 conditional KO mice (Havcr2^cKO^) generated previously via Cre/lox-based gene deletion (23) to identify the cell types relevant for the anti–TIM-3 Ab–mediated effects. To test the role of TIM-3 for alloantigen-driven Tc activation, Tc were exposed in vitro to allogeneic stimulating Tc-depleted PBMCs. We observed that specific TIM-3 deletion in CD8^+^ Tc (*Havcr2^fl/fl^;E8i^cre/+^*) caused increased proliferation compared with *Havcr2^fl/fl^* Tc (Figure 4A). Conversely, TIM-3 deletion in all DCs (*Havcr2^fl/fl^;Cd11c^cre/+^*) or cDC1 (*Havcr2^fl/fl^;Zbtb46^cre/+^*) subsets used for allogeneic stimulation did not affect Tc proliferation (Supplemental Figure 6, A and B). Additionally, upon coculture with allogeneic stimulating cells, *Havcr2^fl/fl^;E8i^cre/+^* Tc exhibited elevated expression of activation markers CD25 and CD69 compared with *Havcr2^fl/fl^* Tc (Figure 4, B–E), which is consistent with the increased proliferation of CD8^+^ Tc. In contrast, TIM-3 deficiency in the stimulating cells did not induce CD25 and CD69 upregulation in Tc (Supplemental Figure 6, C–F).

To analyze the contribution of recipient DCs, we pretreated the mice with anti–TIM-3 Ab at day –4 and day –1 before allo-HCT. The Ab would bind to the recipient’s antigen-presenting cells (APCs) and therefore induce blockade of TIM-3 on these. However, we found no survival advantage in mice pretreated with anti–TIM-3 Ab before allo-HCT (Supplemental Figure 6G), confirming our hypothesis that anti–TIM-3 Ab treatment acts on allogeneic Tc to reinvigorate CD8^+^ Tc and thus reduce Tc exhaustion.

Consistent with enhanced activation, IFN-γ production was increased in *Havcr2^fl/fl^;E8i^cre/+^* Tc compared with *Havcr2^fl/fl^* Tc upon stimulation with allogeneic APCs in vitro for 2 days (Figure 4F) or 4 days (Figure 4G). Next, we aimed to investigate Tc function in vivo by injecting both TIM-3 cKO (Havcr2^cKO^, C57BL/6) BM and Tc into WEHI-3B–bearing allogeneic BALB/c recipient mice. We observed that transfer of Tc caused improved survival of AML-bearing mice after allo-HCT when TIM-3 was deleted in CD8^+^ Tc compared with *Havcr2^fl/fl^* Tc (Figure 4H). Moreover, using *Havcr2^fl/fl^;Cd4^cre/+^* mice as donors for allogeneic BM and Tc led to improved survival of AML-bearing mice after allo-HCT compared with *Havcr2^fl/fl^* Tc (Figure 4I). Supporting our findings in vivo, *Havcr2^fl/fl^;Cd4^cre/+^* Tc exhibited higher cytotoxic capacity against WEHI-3B cells compared with *Havcr2^fl/fl^* Tc in vitro (Figure 4J).

We analyzed the phenotype of Tc from the spleens of *Havcr2^cKO^* mice under steady-state conditions and observed no difference with respect to CD4^+^ and CD8^+^ Tc frequencies (Supplemental Figure 7, A and B) and phenotype (Supplemental Figure 7C) among the 3 different genotypes. We assessed the development of acute graft-versus-host disease (aGVHD) by histological scoring of the liver, small intestines (SI), and colon and found no significant difference between mice that had received *Havcr2^fl/fl^;E8i^cre/+^* or *Havcr2^fl/fl^;Cd4^cre/+^* compared with *Havcr2^fl/fl^* Tc (Supplemental Figure 8, A–C). Additionally, we found a reduction of the leukemia burden in allo-HCT mice that had received *Havcr2^fl/fl^;E8i^cre/+^* or *Havcr2^fl/fl^;Cd4^cre/+^* Tc compared with *Havcr2^fl/fl^* Tc (Supplemental Figure 8D). We also observed a reduced frequency of TOX^+^ cells and an increased frequency of transcription factor 7 (TCF-7^+^) cells among CD8^+^ Tc in allo-HCT mice that had received Tc isolated from *Havcr2^fl/fl^;E8i^cre/+^* or *Havcr2^fl/fl^;Cd4^cre/+^* mice compared with *Havcr2^fl/fl^* mice (Supplemental Figure 8, E–G). TCF-7 is a transcription factor known to be critical for the generation of the CD8^+^ Tc memory response (39). Accordingly, we observed a significant expansion of phenotypic CD8^+^ effector memory Tc (Tem), as seen by the increased frequency of IFN-γ^+^TNF-α^+^ cells among TCF-7^+^CD8^+^ Tc in allo-HCT mice that had received *Havcr2^fl/fl^;E8i^cre/+^* or *Havcr2^fl/fl^;Cd4^cre/+^* Tc compared with *Havcr2^fl/fl^* Tc (Supplemental Figure 8H). These results suggest a more efficient and sustainable CD8^+^ Tc immune response, which could explain the benefit in the survival outcomes observed when TIM-3 is deleted in all donor Tc compared with the deletion of TIM-3 in CD8^+^ Tc only.

Overall, deleting TIM-3 in both CD4^+^ and CD8^+^ Tc has an additive effect compared with deletion in CD8^+^ Tc alone, potentially because CD4^+^ Tc support CD8^+^ Tc function by producing IFN-γ. TIM-3 may inhibit CD4^+^ Tc activity. Conversely, deletion of TIM-3 in CD4^+^ Tc may lead to their consecutive activation, which may further enhance CD8^+^ Tc effector function against AML cells.

Taken together, these results indicate that genetic deletion of TIM-3 in CD8^+^ Tc enhances Tc activation, proliferation, and IFN-γ production in vitro. In vivo deletion of TIM-3 in CD8^+^ Tc or all Tc improves GVL effects. Conversely, TIM-3 deletion in DCs did not affect allogeneic Tc responses in vitro.

### Single-cell RNA-Seq reveals that genetic TIM-3 deletion in CD8^+^ Tc expands CD8^+^ stem-like Tc and promotes features of Teff.

To decipher the mechanism underlying the enhanced GVL effect seen when deleting TIM-3 in CD8^+^ Tc, we performed single-cell RNA-Seq (scRNA-Seq) on donor *Havcr2^fl/fl^* and *Havcr2^fl/fl^;E8i^cre/+^* Tc isolated from the spleen of WEHI-3B–bearing BALB/c recipient mice. We prepared scRNA-Seq libraries from CD3^+^ Tc for 4 samples (*n* = 2 *Havcr2^fl/fl^* and *n* = 2 *Havcr2^fl/fl^;E8i^cre/+^*). Data integration revealed 21 distinct clusters (Supplemental Figure 9A) before exclusion of non-CD3^+^ cells (Supplemental Figure 9B). The analysis was then subclustered into CD4^+^ and CD8^+^ Tc (Supplemental Figure 9C).

**Figure 5:**
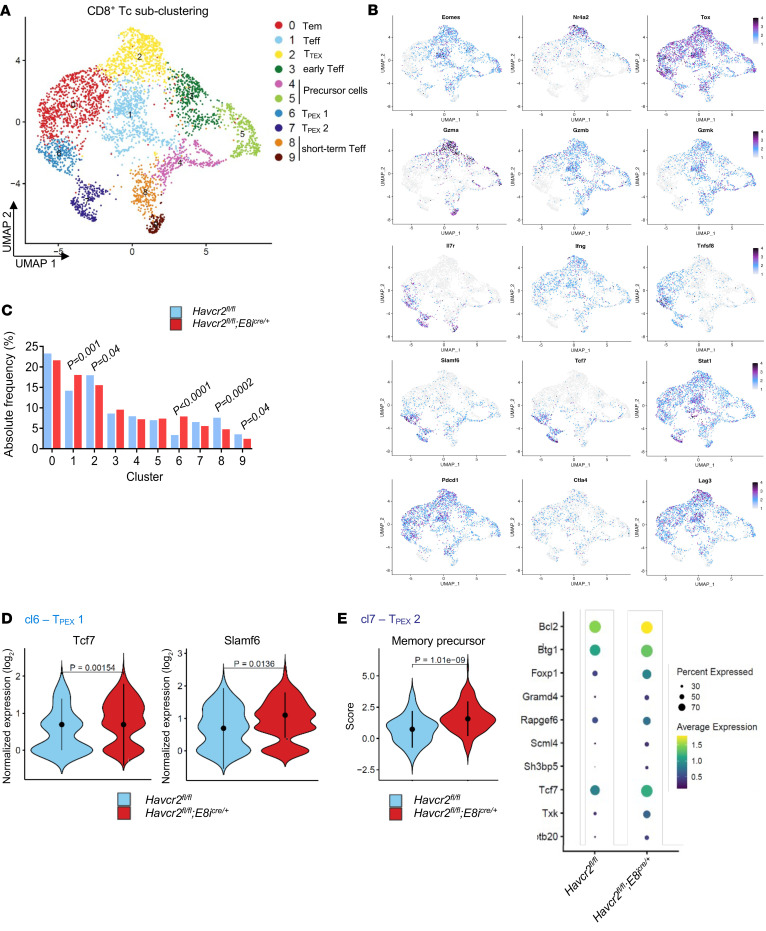
Deletion of TIM-3 in CD8^+^ Tc leads to T_PEX_ expansion. (**A**–**E**) BALB/c recipient mice were injected i.v. with WEHI-3B AML cells (BALB/c background) and 5 × 10^6^ allogeneic *Havcr2^fl/fl^* (*n* = 2) and *Havcr2^fl/fl^;E8i^cre/+^* (*n* = 2) BM and Tc. Tc were isolated at day 23 and stained with an oligo-tagged H-2Kb (donor) Ab allowing scRNA-Seq analysis of FACS-sorted donor Tc. (**A**) UMAP visualization of 10 clusters of CD8^+^ Tc. (**B**) Feature plots showing the expression levels of different marker genes relevant for the characterization of the respective cluster. (**C**) Bar diagram representing the frequency of the different CD8^+^ Tc clusters. Adjusted *P* values were calculated using Fisher’s test. (**D**) Expression levels of key genes differentially expressed in Tc from AML-bearing mice receiving *Havcr2^fl/fl^* (left) and *Havcr2^fl/fl^;E8i^cre/+^* (right) BM/Tc in cluster 6. (**E**) Score of the functional signature enriched in Tc from AML-bearing mice receiving *Havcr2^fl/fl^;E8i^cre/+^* BM/Tc compared with *Havcr2^fl/fl^* BM/Tc in cluster 7. Gene expression analysis of genes within the “memory precursor” signature.

Clustering analysis of CD8^+^ Tc revealed 10 populations of Tc, including 2 clusters of progenitor cells (clusters 4 and 5), 5 subsets of Teff (clusters 0, 1, 3, 8, and 9), and 3 populations of exhausted Tc (clusters 2, 6, and 7) (Figure 5A). The top 6 genes in each cluster of CD8^+^ Tc are shown in Supplemental Figure 10A. All clusters of exhausted Tc expressed characteristic genes, including those encoding for TOX (40), PD-1, and lymphocyte-activation gene 3 (LAG-3). However, in contrast with cluster 6 and cluster 7, cells in cluster 2 also expressed high levels of the genes encoding for the transcription factors eomesodermin (Eomes) and nuclear receptor 4A2 (Nr4a2), which have been reported to be strongly associated with exhaustion (41). Moreover, cluster 6 and cluster 7 were the only clusters to express transcription factor 7 (Tcf7) and the signaling lymphocytic activation molecule family member 6 (Slamf6), allowing us to identify cluster 6 and 7 as precursor exhausted (T_PEX_) and cluster 2 as terminally exhausted (T_TEX_) CD8^+^ Tc (Figure 5B and Supplemental Figure 10A). T_PEX_ are non–fully functional CD8^+^ Tc displaying a stem-like profile that persist long term and differentiate into the T_TEX_ subset. Consequently, T_PEX_ have been described as better controlling tumor growth than T_TEX_ in several solid cancers. Additionally, it has been shown that Tcf7 and Slamf6 expressing CD8^+^ Tc contribute to ICI-mediated antitumor immune responses (42–44). All clusters were represented in both *Havcr2^fl/fl^* and Havcr2^fl/fl^;E8i^cre/+^ conditions, albeit in different proportions (Figure 5C and Supplemental Figure 10, B and C), and the numbers of cells that were present in each CD8^+^ Tc cluster are shown in Supplemental Table 1.

In particular, cluster 6 containing T_PEX_ cells was more abundant in *Havcr2^fl/fl^;E8i^cre/+^* Tc compared with *Havcr2^fl/fl^* Tc. In contrast, cluster 2, which represents T_TEX_ cells, was less abundant in *Havcr2^fl/fl^;E8i^cre/+^* Tc. Additionally, cluster 8 and 9, identified as short-term effector CD8^+^ Tc, were less abundant in *Havcr2^fl/fl^* Tc (Figure 5C). Moreover, Tc in cluster 6 expressed higher levels of Tcf7 and Slamf6 in *Havcr2^fl/fl^;E8i^cre/+^* Tc (Figure 5D), and cluster 7 was strongly enriched for a transcriptomic signature of memory precursor Tc (Figure 5E), indicating an enriched T_PEX_ signature for these 2 clusters of CD8^+^ Tc in *Havcr2^fl/fl^;E8i^cre/+^* Tc. Additionally, we performed analysis of gene-set enrichment in CD8^+^ Tc clusters, revealing significant transcriptional differences between *Havcr2^fl/fl^;E8i^cre/+^* and *Havcr2^fl/fl^* groups (Supplemental Figure 10D). In particular, T_PEX_ subsets in the *Havcr2^fl/fl^;E8i^cre/+^* group showed enrichment in the “IL-2/Stat5 signaling pathway” and “inflammatory response.” Notably, cluster 7 (T_PEX_ subset) was enriched for the “Wnt/β-catenin signaling” pathway, crucial for Tc differentiation, effector functions, and migration (45). Furthermore, several studies have described the importance of Wnt/β-catenin signaling in the activation and maintenance of T_PEX_, which display indispensable antitumor capacities (46, 47), confirming an enriched signature for memory Tc in the *Havcr2^fl/fl^;E8i^cre/+^* group. These results demonstrate that TIM-3 deletion in CD8^+^ Tc promotes the expansion of polyfunctional T_PEX_ and effector Tc, contributing to the enhanced GVL effects observed in mice receiving *Havcr2^fl/fl^;E8i^cre/+^* Tc compared with those receiving *Havcr2^fl/fl^* Tc.

Within CD4^+^ Tc subclusters, we identified 6 distinct cell types, including a Treg cluster (cluster 5), and 5 clusters of effector/memory CD4^+^ Tc (Supplemental Figure 11, A and B). Clusters 0 and 2, containing Tem/Teff, were more abundant in TIM-3–deficient CD8^+^ Tc. Cluster 4, identified as resident-memory–like CD4^+^ Tc, was more abundant in *Havcr2^fl/fl^* Tc (Supplemental Figure 11C). Cluster 1, characterized by high Tcf7 and Slamf6 expression, described precursor effector memory Th1 cells, required for long-term and heightened antitumor immunity (48), including in the context of allo-HCT (49). We found that cells in cluster 1 exhibited an enriched transcriptomic signature for memory precursor Tc in the *Havcr2^fl/fl^;E8i^cre/+^* group (Supplemental Figure 11D). Additionally, cluster 3 was strongly enriched for a transcriptomic signature of early activated cytotoxic CD4^+^ Tc in *Havcr2^fl/fl^;E8i^cre/+^* Tc (Supplemental Figure 11E). The top 6 genes in each cluster of CD4^+^ Tc is shown in Supplemental Figure 12A. All clusters were represented in both *Havcr2^fl/fl^* and *Havcr2^fl/fl^;Cd4^cre/+^* Tc (Supplemental Figure 11C and Supplemental Figure 12, B and C), and the numbers of cells that are present in each CD4^+^ Tc cluster are shown in Supplemental Table 1. Additionally, analysis of gene-set enrichment in CD4^+^ Tc clusters revealed enriched signatures for interferon responses and an enriched “inflammatory response” pathway in the *Havcr2^fl/fl^;E8i^cre/+^* group, indicating a more efficient immune response (Supplemental Figure 12D). These results demonstrate that TIM-3 deletion in CD8^+^ Tc contributes to the expansion of precursor effector memory Th1-like and early activated cytotoxic CD4^+^ Tc subsets, highlighting TIM-3’s complex role in Tc subpopulations.

### Anti–TIM-3 treatment results in immune recall against AML cells without enhancing aGVHD.

**Figure 6:**
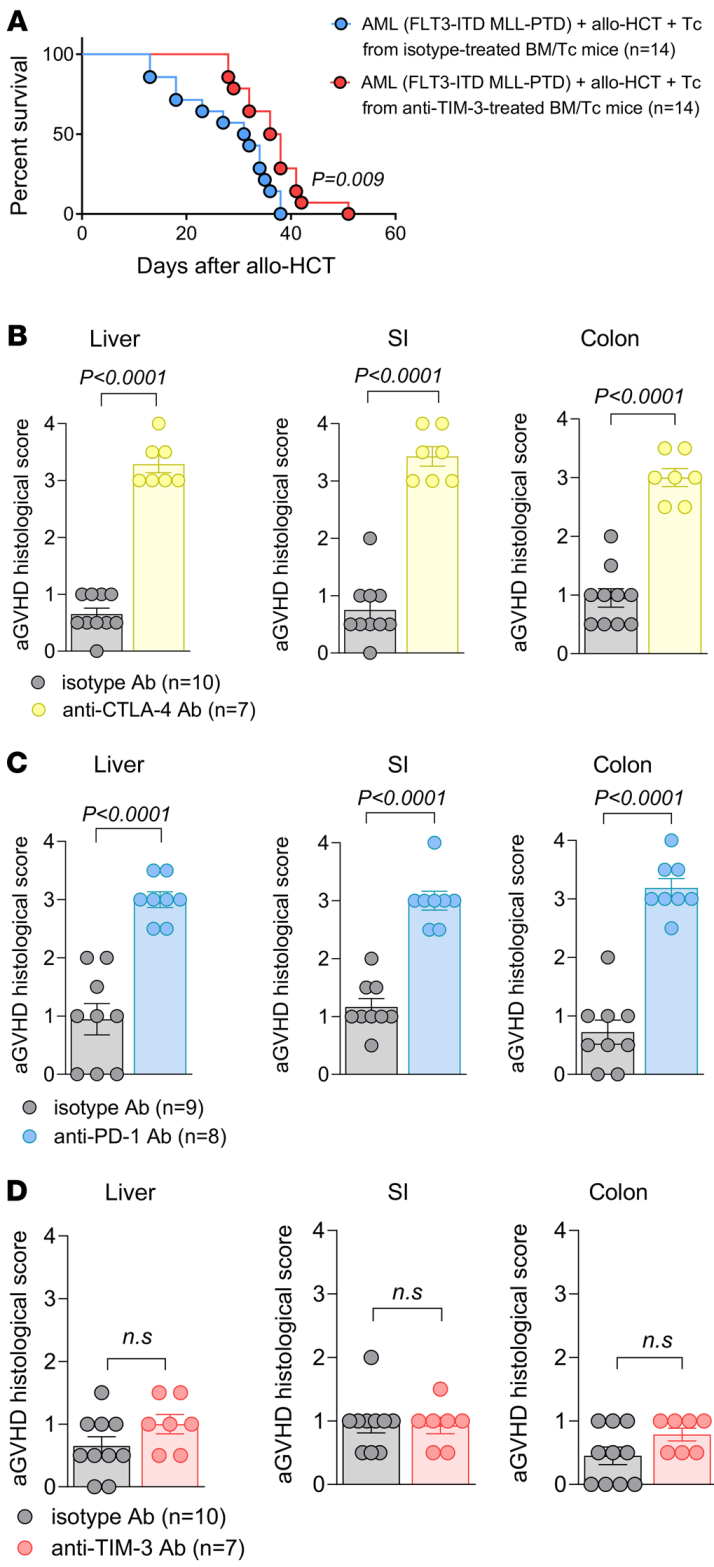
Anti–TIM-3 treatment leads to recall immunity against AML cells without inducing aGVHD. (**A**) Kaplan-Meier plot showing mouse survival in the indicated groups. C57BL/6 recipient mice were injected with FLT3-ITD MLL-PTD AML cells and 5 × 10^6^ allogeneic BM. At day 2, mice received adoptive transfer of 3 × 10^5^ allogeneic donor Tc. Allogeneic Tc were isolated on day 15 from FLT3-ITD MLL-PTD AML–bearing mice, which received allogeneic BM/Tc and were treated with isotype (*n* = 14) or anti–TIM-3 (*n* = 14) Ab. (**B**–**D**) The scatter plots show the histopathological aGVHD severity in the indicated groups. BALB/c recipient mice were injected i.v. with 5 × 10^6^ allogeneic BM and 4 × 10^5^ allogeneic Tc. From day 1 to day 5, mice were treated (150 μg, i.p.) with anti–TIM-3/isotype, anti–PD-1/isotype, or anti–CTLA-4 isotype. aGVHD histological scores were determined on liver, SI, and colon at day 7 for the different groups. Data are represented as mean ± SEM from 2 independent experiments. *P* values were calculated using an unpaired Student’s *t* test.

We assessed the potential of TIM-3 Ab treatment to induce immune memory against AML cells by isolating donor Tc from AML-bearing mice after allo-HCT treated with anti–TIM-3 or isotype Abs. These Tc were transferred into secondary AML-bearing mice after allo-HCT, resulting in improved survival in mice that received Tc from anti–TIM-3–treated recipients compared with isotype-treated mice (Figure 6A).

To evaluate the impact of anti–TIM-3 treatment on aGVHD, we examined SI, colon, and liver tissues from mice treated with anti–PD-1, anti–cytotoxic T lymphocyte–associated protein 4 (anti–CTLA-4), or anti–TIM-3 Ab after allo-HCT. All Abs (150 μg) were given daily, from day 1 to day 5. Histopathological analysis revealed increased aGVHD severity in all organs from mice treated with anti–PD-1 or anti–CTLA-4 compared with their respective isotype Ab treatment (Figure 6, B and C). Conversely, anti–TIM-3 treatment did not exacerbate aGVHD severity in these organs compared with the isotype Ab (Figure 6D).

### The clinical-grade humanized anti–TIM-3 Ab sabatolimab enhances the GVL effect.

**Figure 7:**
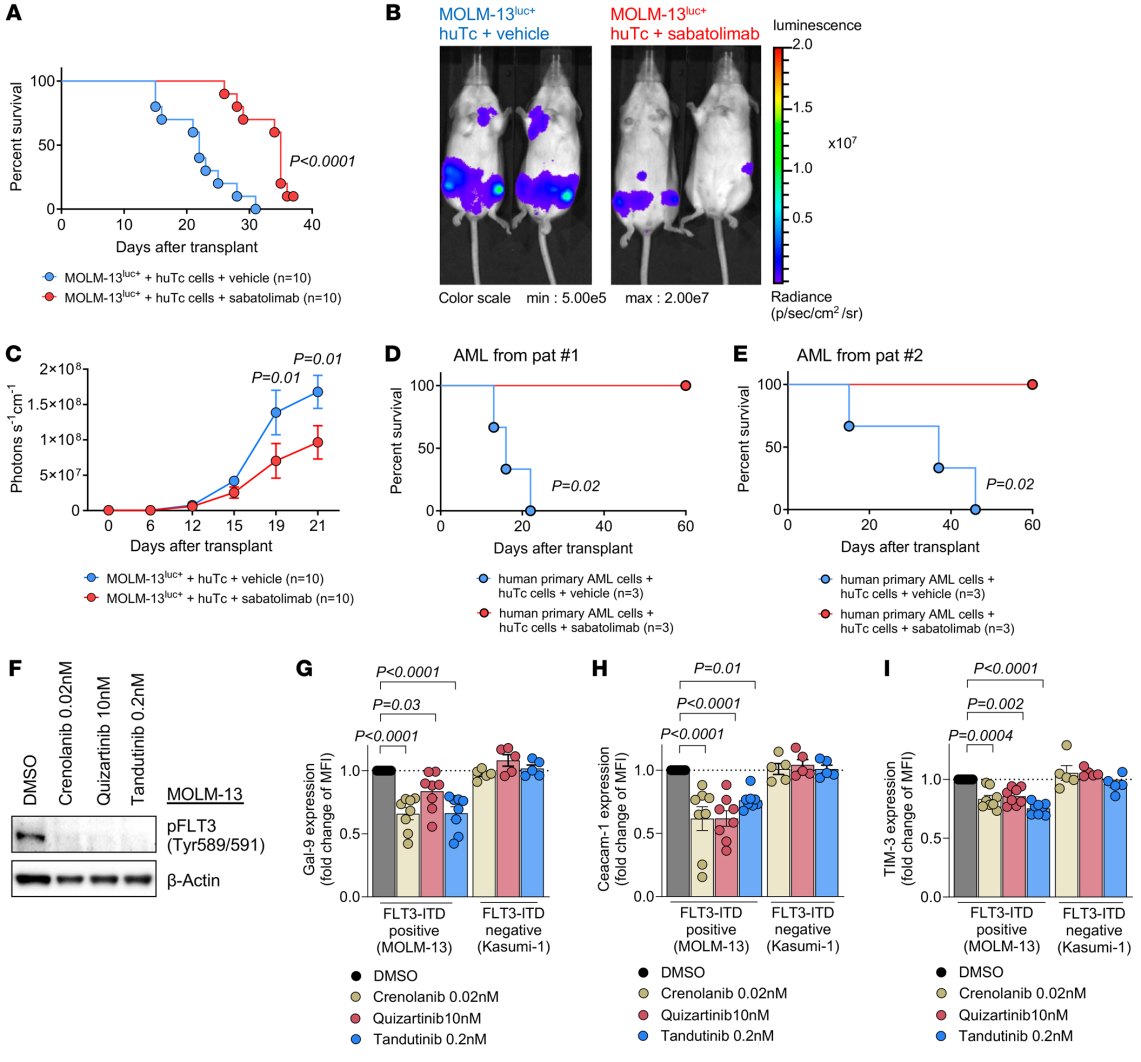
Anti-human TIM-3 Ab sabatolimab enhances the GVL effect. (**A**) Kaplan-Meier plots showing mouse survival in the indicated groups. *Rag2^–/–^Il2rg^–/–^* recipient mice were injected i.v. with human-derived MOLM-13^luc+^ AML cells and human CD3^+^ Tc (from HLA-nonmatched healthy donors) and treated with vehicle (*n* = 10) or human anti–TIM-3 (*n* = 10). Results show 3 independent experiments, and *P* value was calculated using 2-sided Mantel-Cox test. (**B**) Representative images of bioluminescence imaging (BLI) of MOLM-13^luc+^ AML–bearing *Rag2^–/–^Il2rg^–/–^* recipient mice 21 days after injection of AML cells, following human Tc injection and vehicle or human anti–TIM-3 Ab treatment. (**C**) BLI signal quantification shows the expansion of AML cells over time. Data are represented as mean ± SEM from 3 independent experiments using 3 different healthy Tc donors. *P* values were calculated using 2-sided Mann-Whitney *U* test. (**D** and **E**) Kaplan-Meier plots showing mouse survival in the indicated groups. *Rag2^–/–^Il2rg^–/–^* recipient mice were injected i.v. with CD3-depleted primary AML cells (from PB at primary diagnosis) and treated with vehicle (*n* = 3) or human anti–TIM-3 (*n* = 3). Each survival curve represents 1 individual AML donor patient. *P* values were calculated using 2-sided Mantel-Cox test. (**F**) Representative Western blots showing the inhibition of phosphorylated FLT3 (pFLT3) (Tyr589/591) upon treatment with 3 different FLT3 inhibitors and loading control (β-actin) in MOLM-13 cells (FLT3-ITD). (**G**) Gal-9, (**H**) CEACAM1, and (**I**) TIM-3 protein expression as relative MFI was determined by FC upon the treatment with the indicated FLT3 inhibitors in FLT3-ITD–positive human cell line (MOLM-13) or FLT3 WT human cell line (Kasumi-1). Fold change is calculated in comparison with the treatment using DMSO (control treatment). Data are represented as mean ± SEM from 8 independent experiments using MOLM-13 cells and *n* = 5 independent experiments using Kasumi-1 cells. *P* values were calculated using ordinary 1-way ANOVA.

To further explore the potential of TIM-3 blockade in improving GVL effects against human leukemia, we used a humanized mouse model. Immunodeficient *Rag2^–/–^Il2rg^–/–^* mice were injected with the human FLT3-ITD mutant AML cell line MOLM-13^luc+^ and allogeneic human Tc (18). These *Rag2^–/–^Il2rg^–/–^* mutant mice lack T, B, and NK cells and are therefore suitable hosts for transplanted human immune cells. Mice were treated i.p. with 150 μg of the humanized anti–TIM-3 Ab (MBG453) or vehicle every 3 days from day 7 to day 28. Anti–TIM-3 Ab treatment resulted in improved survival (Figure 7A) and reduced leukemia burden, as assessed on day 21 after allo-HCT by luciferase imaging (Figure 7, B and C) compared with vehicle-treated mice. Additionally, *Rag2^–/–^Il2rg^–/–^* mice receiving CD3-depleted human primary AML blasts and donor Tc from HLA-mismatched healthy donors and treated with sabatolimab showed improved survival (Figure 7, D and E). We also investigated whether FLT3-ITD mutational status affects TIM-3. We treated human-derived MOLM-13 cells with different FLT3 inhibitors (crenolanib, quinzartinib, and t andutinib) to prevent FLT3 signaling and observed a decreased expression of Gal-9, CEACAM1, and TIM-3 in FLT3-ITD mutant AML cells, but not in Kasumi-1 cells (FLT3 WT) (Figure 7, F–I), indicating that oncogenic FLT3-ITD signaling upregulates TIM-3 and its ligands in human AML.

### Expression of TIM-3 and its ligands in different cell populations isolated from patients with AML relapse after allo-HCT.

**Figure 8:**
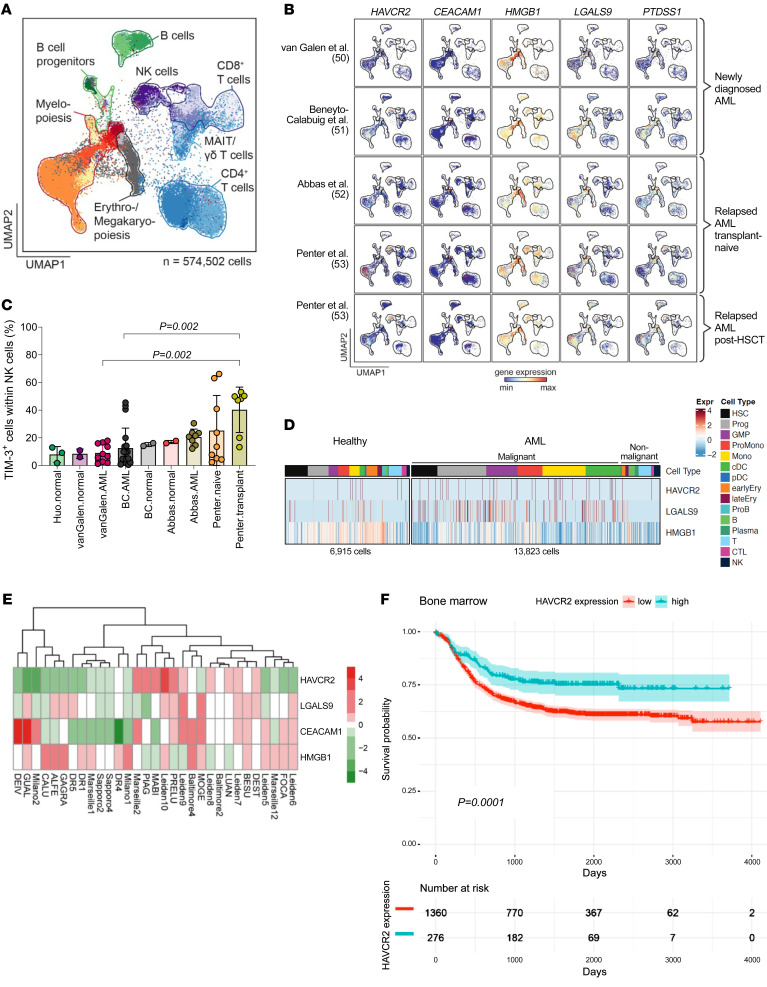
Expression of TIM-3 and its ligands in human primary samples. (**A**) Overview of UMAP embedding colored by 27 projected cell types of 574,502 scRNA-Seq profiles from van Galen et al. (50) (*n* = 20,362), Abbas et al. (52) (*n* = 127,027), Huo et al. (54) (*n* = 20,385), Beneyto-Calabuig et al. (51) (*n* = 101,767), and Penter et al. (53) (*n* = 304,961). Major cell types are highlighted. (**B**) Scaled gene expression of *HAVCR2*, *CEACAM1*, *HMGB1*, *LGALS9*, and *PTDSS1* across AML BM scRNA-Seq profiles. AML BM cases include newly diagnosed and relapsed disease with or without allo-HCT. (**C**) Proportion of TIM-3^+^ cells of all NK cells across normal and AML BM data sets. (**D**) Heatmap generated from scRNA-Seq profiles from van Galen et al. (50) showing expression of *HAVCR2*, *LGALS9*, and *HMGB1* in normal hematopoiesis and AML cell types of patients at diagnosis of AML. (**E**) Expression of *HAVCR2*, *LGALS9*, *CEACAM1*, and *HMGB1* in human AML cells determined by bulk RNA-Seq. AML cells were isolated from patients with AML relapse after allo-HCT at multiple transplant centers. (**F**) Probability of survival stratified according to high versus low *HAVCR2* gene expression in AML BM at diagnosis within the target-AML cohort (58). RNA-Seq data were derived from the GenomicDataCommons (GDC) library (https://gdc.cancer.gov/). Gene expression more than 1 SD above mean was defined as high expression.

To clarify whether our results in mice could be related to the human context, we evaluated the expression of TIM-3 (*HAVCR2*) and its ligands (*CEACAM1*, *HMGB1*, *LGALS9*, and *PTDSS1*) in primary patient samples. First, we interrogated published scRNA-Seq data sets of human BM samples in the setting of newly diagnosed AML (50, 51), transplant-naive (52, 53) or posttransplant AML relapse (53), and physiologic hematopoiesis (50–52, 54). To enable comparison across these data sets, we annotated hematopoietic and immune cells based on a healthy BM reference (see Methods) (55) (Figure 8A). In normal hematopoiesis and in AML, *HAVCR2* expression was detectable in myeloid progenitor cells, Tc, and NK cells (56), and all 4 TIM-3 ligands were expressed by HSC and progenitor populations (Figure 8B and Supplemental Figure 13A). We found high *HAVCR2* expression in NK cells, in agreement with previous reports (56), which might be particularly relevant for the early posttransplant context, where NK cells are more abundant than in transplant-naive AML (Figure 8C). Additionally, it has been shown that TIM-3 is highly expressed by NK cells in patients with AML, correlating with enhanced effector functions and NK cell cytotoxicity and improved clinical outcome in AML patients (57). Compared with healthy myelopoiesis, we observed higher expression of *HAVCR2* and *LGALS9* but not *HMGB1* in newly diagnosed AML (Figure 8D and Supplemental Figure 13A). Expression of CEACAM1 was found below the detection limit and only detected in 0.4% of the cells (data not shown) (50). Second, we evaluated the expression of TIM-3 and its ligands in human primary AML cells isolated from patients that experienced a relapse after allo-HCT at multiple transplant centers (Supplemental Table 2) using bulk RNA-Seq. We observed heterogeneous expression of *HAVCR2* and its ligands *LGALS9*, *CEACAM1*, and *HMGB1* (Figure 8E) as well as variable counts of TIM-3^+^ Tc (cells/μL) in peripheral blood (PB) of patients 1, 2, or 3 months after allo-HCT (Supplemental Figure 13B). These results may suggest differential therapeutic sensitivity to TIM-3 inhibition.

To understand whether TIM-3 expression affects survival, we analyzed the probability of survival depending on the level of expression of *HAVCR2* (mean) in bulk RNA-Seq data derived from published databases on human cells. We defined *HAVCR2* gene expression more than 1 SD above mean as high expression. We observed that patients with high *HAVCR2* expression in BM cells at diagnosis showed significantly improved survival compared with patients with low *HAVCR2* expression when analyzing the target-AML cohort (58), comprising 1,630 low and 276 high TIM-3–expressing BM patient samples (Figure 8F). This pattern was also observed in PB (Supplemental Figure 14A). The improved survival with higher TIM-3 expression may be linked to a high count of TIM-3^+^ NK cells, which have the highest TIM-3 levels.

## Discussion

Since relapse of the underlying malignancy is the major cause of death beyond day 100 after allo-HCT (59), novel strategies for preventing or treating relapse are urgently needed. Tc exhaustion and increased immune checkpoint ligand expression have been reported in patients with AML relapse after allo-HCT (3, 30), which supports the use of approaches that target exhausted Tc populations. Prior studies have shown that targeting immune checkpoint molecules using anti–PD-1 and anti–CTLA-4 Ab have clinical activity in a fraction of AML patients after allo-HCT; however, aGVHD has limited the use of these Ab in the patients (13, 14, 60).

In this study, we deciphered the potential of targeting TIM-3 to overcome immune escape and reinvigorate immune cells to enhance antileukemia immunity after allo-HCT in AML.

We observed that anti–TIM-3 Ab treatment reduced features of Tc exhaustion and enhanced Tc effector functions, leading to improved GVL activity in multiple murine and humanized AML models. Specific TIM-3 deletion on CD8^+^ Tc allowed us to dissect the mechanism underlying the improvement of GVL effects. We identified 2 subpopulations of CD8^+^ exhausted Tc and found that polyfunctional stem-like T_PEX_ cells expanded when TIM-3 was deleted in CD8^+^ Tc while cytotoxic but short-lived T_TEX_ population remained unchanged, leading to a more effective, robust, and durable Tc-mediated immune response. TIM-3 deletion in the CD8^+^ Tc subset also affected CD4^+^ Tc subpopulations, in particular causing expansion of Th1-like memory CD4^+^ Tc, recently described as displaying improved survival and persistence functions in the context of alloimmunity (49).

TIM-3 is also highly expressed on NK cells. However, its role in modulating NK function remains unclear, particularly in human diseases. The expression of TIM-3 on NK cells has been reported as being associated with both NK activation (61, 62) and dysfunction/exhaustion (63), depending on the cytokine stimulation (64). We observed an increased frequency of TIM-3–expressing NK cells in early posttransplant AML patients, consistent with other studies reporting that high TIM-3 expression on NK cells from AML patients correlates with an enhanced NK cell cytotoxicity and effector functions and an ultimately improved clinical outcome in AML patients (57).

We also observed a higher frequency of neutrophils within immune cells isolated from leukemia-infiltrated spleens of AML-bearing mice upon anti–TIM-3 Ab treatment, consistent with several recent independent studies showing that neutrophils may contribute to tumor control (36, 37). The authors reported that ICI therapy–exposed neutrophils acquired an IFN-γ gene signature, suggesting their contribution to the antitumor immune responses. Hirschhorn et al. identified a distinct antitumorigenic neutrophil subset that occurred in ICI-treated mice (36). They described the interaction between Tc mediating the initial antitumor immune response and neutrophils that eliminate tumor antigen loss variants (36).

Our study revealed that enhanced antitumor immunity upon anti–TIM-3 Ab therapy involves additional indirect immune mechanisms, recently described upon ICI therapy in solid cancers (36, 37).

A major concern when using ICI after allo-HCT is the development of severe aGVHD reported with anti–PD-1 and anti–CTLA-4 therapy (13–16). Consistently, we found that anti–PD-1 and anti–CTLA-4 treatment induced aGVHD in mice. Conversely, we observed no GVHD enhancement when using anti–TIM-3 Ab treatment. This might be because TIM-3 expression is restricted to terminally differentiated Tc (65, 66), which may contribute less to GVHD. However, the absence of GVHD development may be strictly dependent on the specific anti–TIM-3 Ab and the administration schedule. A previous preclinical study by Veenstra et al. (67) using a TIM-3–Ig fusion protein demonstrated that TIM-3 blockade exacerbated aGVHD in mice. Conversely, we treated the mice with an anti–TIM-3 Ab (monoclonal Ab, clone 5D12). Further, the group showed that TIM-3^−/−^ mice used as donors led to enhanced aGVHD, whereas we used mice carrying a conditional specific TIM-3 deletion in selected immune subpopulations. Additionally, the number of donor Tc used to induce GVHD was 10 times higher (3 × 10^6^ of purified CD25^-^ Tc) compared with our model (3 × 10^5^ pan-Tc). These studies show that usage of TIM-3 Abs requires caution with respect to aGVHD development.

Previous studies suggested that targeting TIM-3 may directly eliminate LSCs via ADCC due to high TIM-3 expression on AML blasts and interruption of the Gal-9 feedback loop by the Ab (25, 26). However, we found that anti–TIM-3 treatment’s effectiveness depended on the presence of alloreactive Tc, with no direct effect on AML cells.

Furthermore, our data suggest that specific oncogenic mutations may influence the effectiveness of ICIs against AML after allo-HCT, with TIM-3 blockade potentially benefiting patients with specific mutations and high TIM-3 ligand expression. Following our previous observation that certain oncogenic mutations lead to enhanced expression of inhibitory checkpoint ligands (29), we found that TIM-3 ligands Gal-9 and CEACAM1 were overexpressed in HSCs carrying FLT3-ITD and c-kit-D816V mutations. Treating mice with mutated AML cells using an anti–TIM-3 Ab resulted in a robust response upon anti–TIM-3 treatment, confirming our hypothesis. This highlights the potential for personalized TIM-3 blockade therapy in AML patients, emphasizing the need for innovative clinical study designs that consider leukemia genetics. Taken together, our findings demonstrate that TIM-3 and its ligands contribute to immune evasion, complementing research showing higher Gal-9/Tim-3 expression in AML patients who fail chemotherapy (27).

We conducted allo-HCT with donor Tc that recognize foreign MHC on AML cells, mimicking the GVL effect observed in patients. However, besides alloantigen-specific immune responses, tumor antigen–specific immune responses that are MHC independent may also play a role. Previous studies have demonstrated the potential to target leukemia cells using leukemia-reactive Tc lines (68).

Our preclinical work in part motivated a phase Ib/II, open-label, multicenter clinical trial using sabatolimab monotherapy for patients with molecular relapse after allo-HCT (EUDRACT number 2020-000869-17, ClinicalTrials.gov NCT04623216); no GVHD has been observed in the 21 patients enrolled in the trial to date (69).

In summary, our study elucidates the link between oncogenic mutations in AML and TIM-3 ligand expression and identifies anti–TIM-3 treatment as a strategy for enhancing the GVL effect through metabolic and transcriptional reprogramming of Tc after allo-HCT. These findings support the ongoing clinical evaluation of sabatolimab for AML patients following allo-HCT.

## Methods

### Sex as a biological variable.

Our study examined male and female animals, and similar findings are reported for both sexes.

### Study design.

The objective of this study was to target TIM-3 to enhance antileukemia immune responses after allo-HCT. We employed well-established allogeneic and xenogeneic GVL and aGVHD murine models to investigate the role of TIM-3 and its ligands. We utilized mouse anti–TIM-3 Ab (clone 5D12) or isotype Ab (mIgG1), clinical grade anti-human TIM-3 Ab (MBG453 Sabatolimab), or vehicle (5% glucose solution) or conditional KO (Havcr2^cKO^) mice. Anti–TIM-3 Ab (5D12 and MBG453) and isotype Ab were provided by Novartis Institutes of Biomedical Research.

All Abs used for FC are summarized in Supplemental Tables 3 and 4.

### Animal models.

C57BL/6 (H-2Kb) and BALB/c (H-2Kd) mice were purchased from Janvier Labs. *Rag2^–/–^Il2rg^–/–^* mice were obtained from the local stock at the animal facility of Freiburg University Medical Center. C57BL/6 Havcr2^cKO^ mice (*Havcr2^fl/fl^*, *Havcr2^fl/fl^;E8i^cre/+^*, *Havcr2^fl/fl^;Cd4^cre/+^*, *Havcr2^fl/fl^;Cd11c^cre/+^* and *Havcr2^fl/fl^;Zbtb46^cre/+^*) were provided by the Gene Lay Institute of Immunology and Inflammation and were described previously (23). Seven- to twelve-week-old mice and female or male donor/recipient pairs were used.

### Cell lines.

WEHI-3B cells (ACC 26), MOLM-13 cells (ACC 554), and 32D cells (ACC 411) were obtained from DSMZ. MOLM-13 cells were then transduced in our lab to express luciferase. Kasumi-1 cells were provided by Michael Lübbert (Freiburg University Medical Center). Cell lines were routinely tested for mycoplasma contamination and authenticated by STR profiling. FLT3-ITD MLL-PTD splenocytes were provided in-house.

### Additional experimental procedures.

All other materials and methods are described in the Supplemental Methods.

### Statistics.

The comparison between quantitative and qualitative variables was done using 2-tailed Student’s *t* test (parametric) or the Mann-Whitney *U* test (nonparametric). Variance analysis was done using a 1-way ANOVA (parametric) or Kruskal-Wallis (nonparametric) test. Cox’s model was applied for survival analysis. For spectral FC analysis, differentially expressed proteins were tested with moderated *t* test of limma. Concerning bar diagrams representing the frequency of the different Tc clusters, adjusted *P* values were calculated using Fisher’s test. Patients were stratified based on high versus low expression of HAVCR2 gene expression in PB. Gene expression more than 1 SD above the mean was considered as high expression. Statistical significance was set at a *P* value of less than 0.05. All statistical analyses used are described in the corresponding figure legends.

### Study approval.

Human PB and/or BM samples were derived from AML patients treated at the Department of Medical Oncology, Dana-Farber Cancer Institute, after prior informed consent. Human PB was collected in sterile EDTA tubes, and PBMCs were isolated using gradient centrifugation (Pancoll Human). Written, informed consent was obtained from each patient, and analysis of human data was carried out in compliance with relevant ethical regulations. The characteristics of patients are listed in Supplemental Table 2. Animal protocols were approved by the animal ethics committee Regierungspräsidium Freiburg, Freiburg, Germany (protocol numbers G23/049, G20/103, G20/75, and G20/096).

### Data availability.

The scRNA-Seq data have been deposited in the NCBI’s Gene Expression Omnibus database (GEO GSE242334). The procedures used for the analysis of human scRNA-Seq data sets are described at https://github.com/petervangalen/ reanalyze-aml2019/blob/main/230830_Heatmaps_Zeiser.R (branch: main; commit ID f9c898b). Values for all data points in graphs are reported in the Supporting Data values file.

## Author contributions

NTB and LMB developed the overall concept of the study, performed the experiments, analyzed all data, generated figures, and wrote the manuscript. NTB and LMB made important contributions, and first authorship order was based on the amount of work each first author contributed to the study. KOD and VKK provided the Havcr2^cKO^ mice and helped to analyze the scRNA-Seq results. MZ helped to perform scRNA-Seq experiments. AH helped to prepare histology slides. TW, LR and BB generated and analyzed spectral FC data. LP, MA, JR, NS, and PVG provided and analyzed human scRNA-Seq data sets. GA, MB, and NK analyzed the scRNA-Seq data. AR collected patient samples, helped with experiments, and helped to write the manuscript. KM reanalyzed published human databases. PA, JMB, NCW, and ELP helped to perform the LC-MS experiments and helped with the analysis. YC and AK helped with the analysis of the metabolomics. PA helped to develop the concept for single-cell FC-based metabolism technology. SD, CK, HE, AA, and JE helped with experiments. MAS, TB, ALI, and JD provided plasmids and helped with experimental design. ASG performed histopathological analyses. HLP helped with experimental design. LV analyzed human data from databases. HDM provided essential reagents (MBG453 sabatolimab). BRB, CJW, RJS, NK, TW, and VKK contributed to critical analysis of the data. RZ developed the overall concept of the study, supervised the experiments, analyzed data, and wrote the first version of the manuscript.

## Supplementary Material

Supplemental data

Supporting data values

## Version 1 06/25/2024

In-Press Preview

## Version 2 08/15/2024

Electronic publication
