# Supplementary material for: Oncogene-induced TIM-3 ligand expression dictates susceptibility to anti–TIM-3 therapy in mice
Source: J Clin Invest. 2024 Jun 25;134(16):e177460. doi: 10.1172/JCI177460 (PMC11324309; doi:10.1172/JCI177460)
Supplement: Supplemental data [file jci-134-177460-s211.pdf]

Suppl. Figure 1

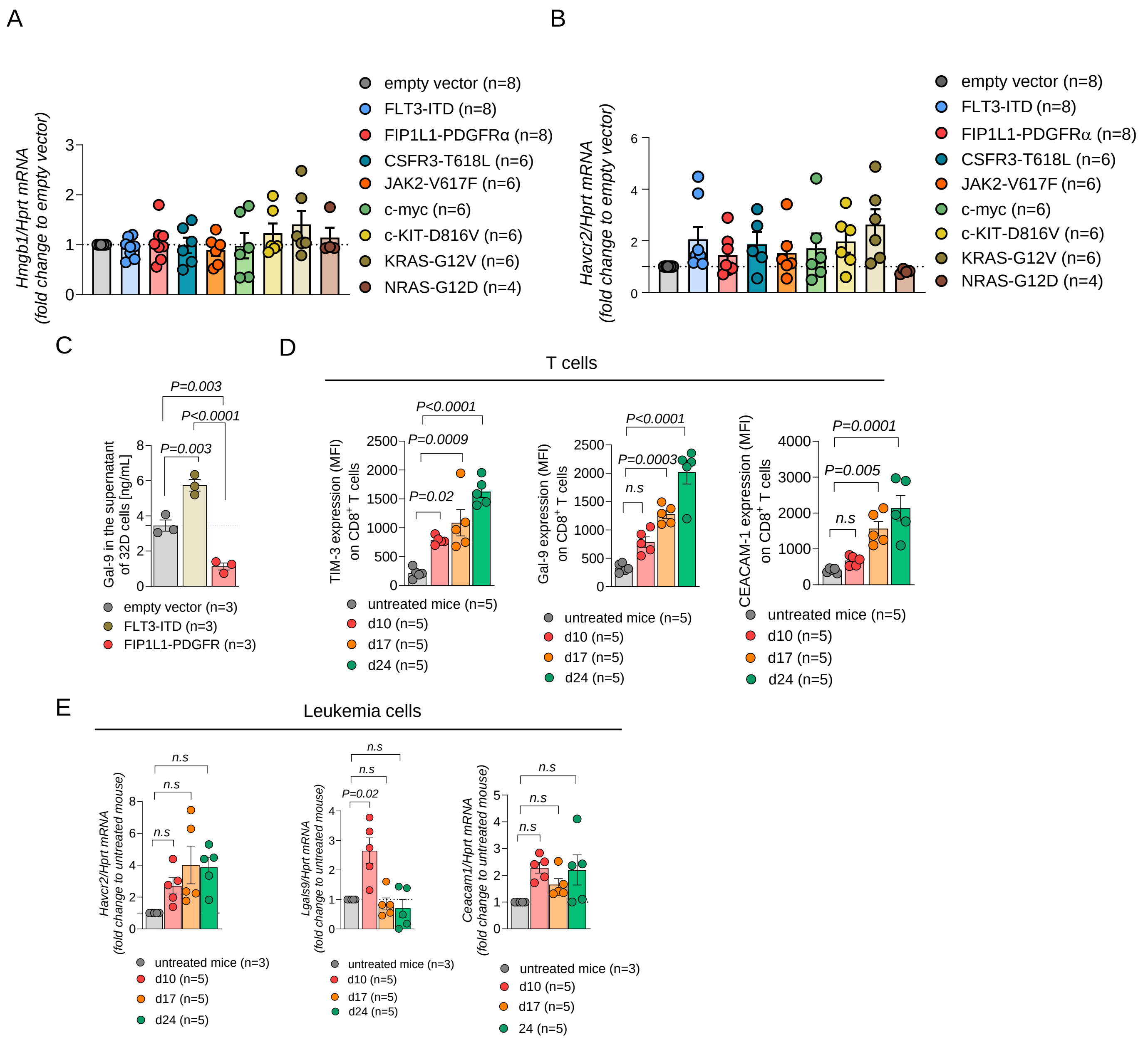

Suppl. Figure 2

A

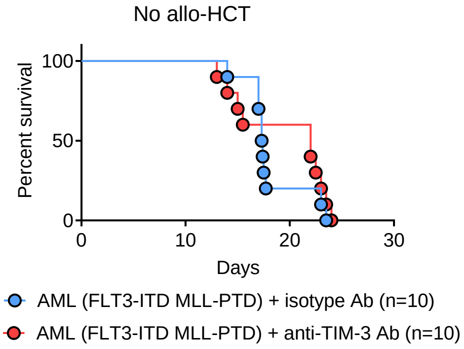

Suppl. Figure 3

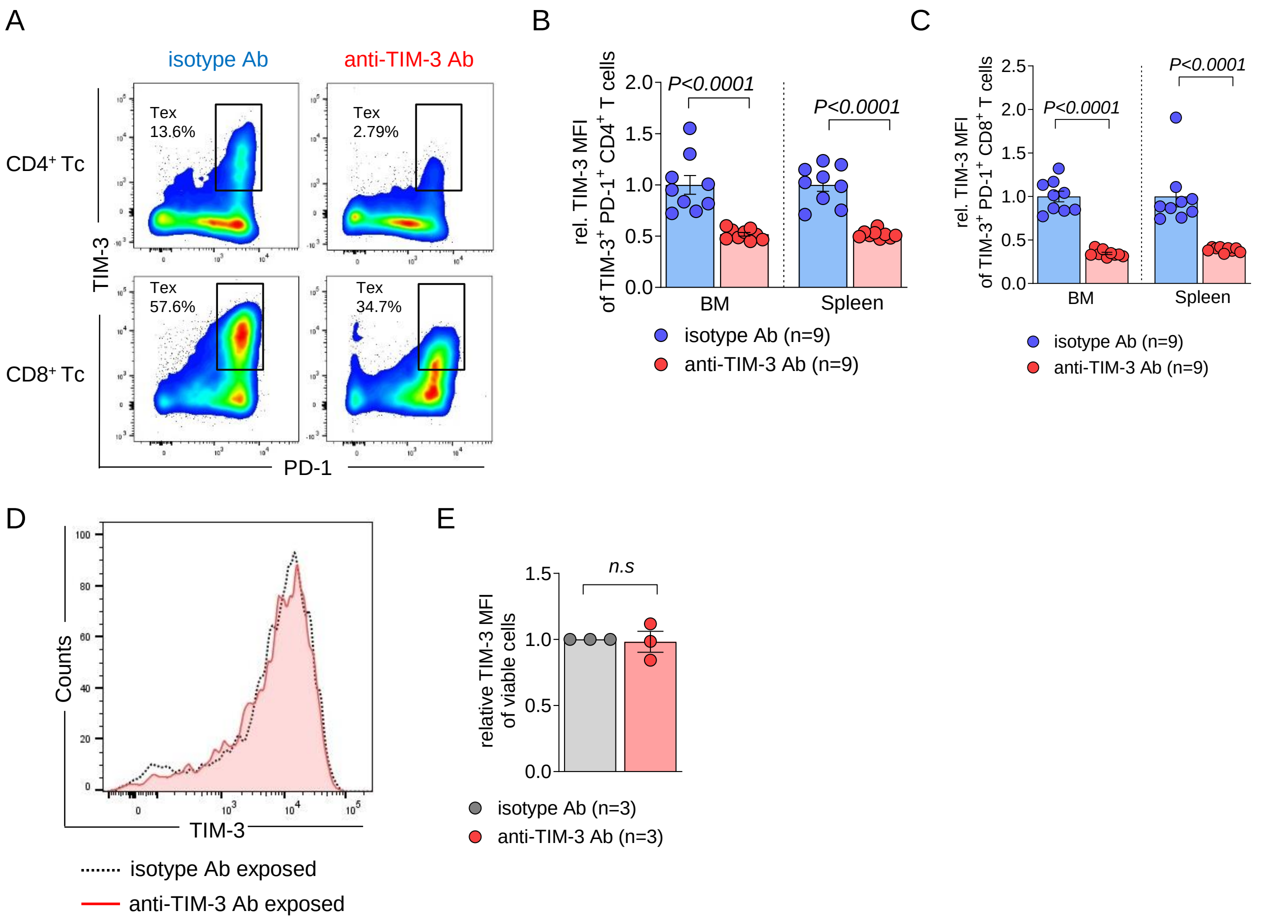

Suppl. Figure 4

A

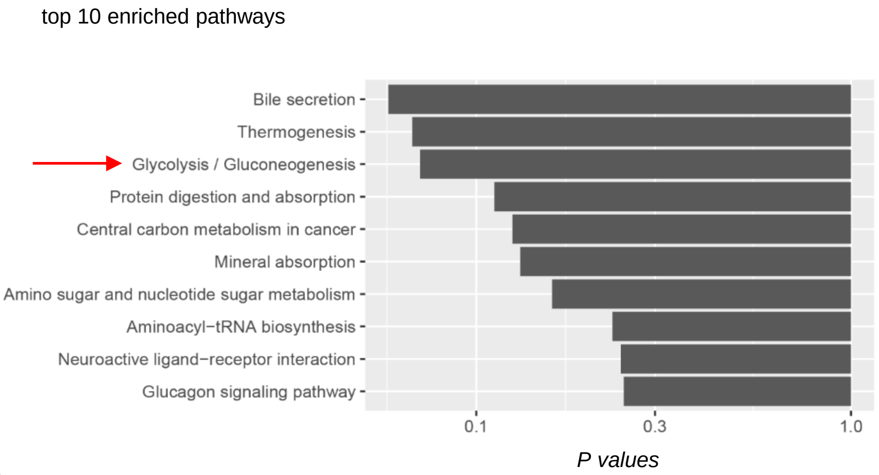

B

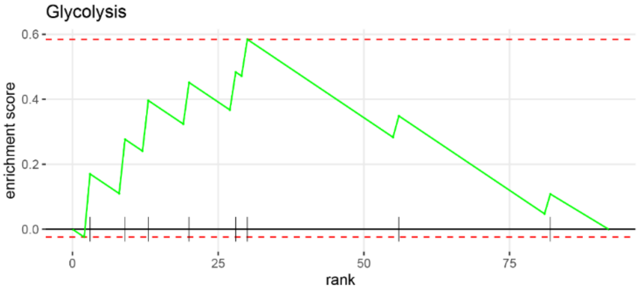

Suppl. Figure 5

A

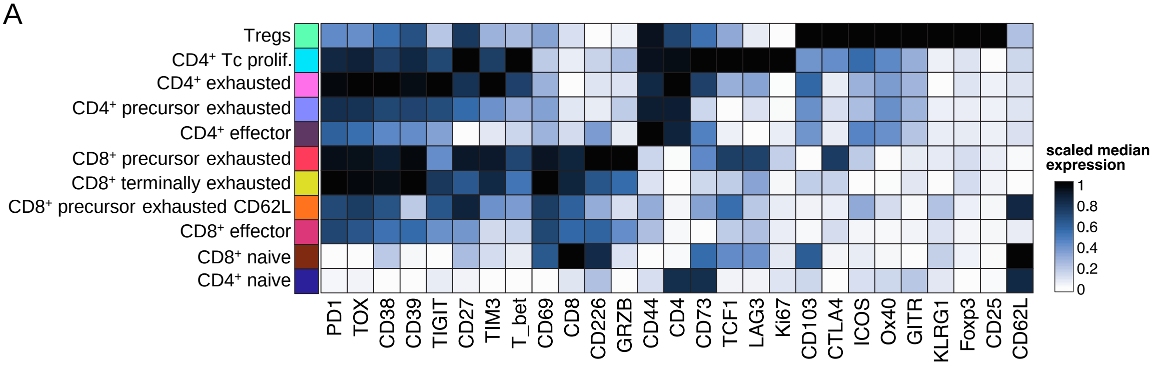

B

Differential expression in myeloid subsets

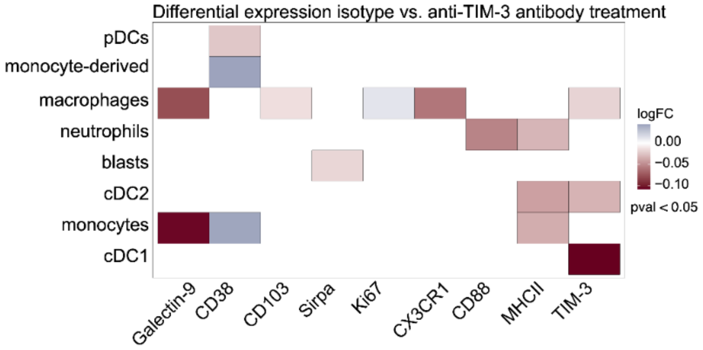

Suppl. Figure 6

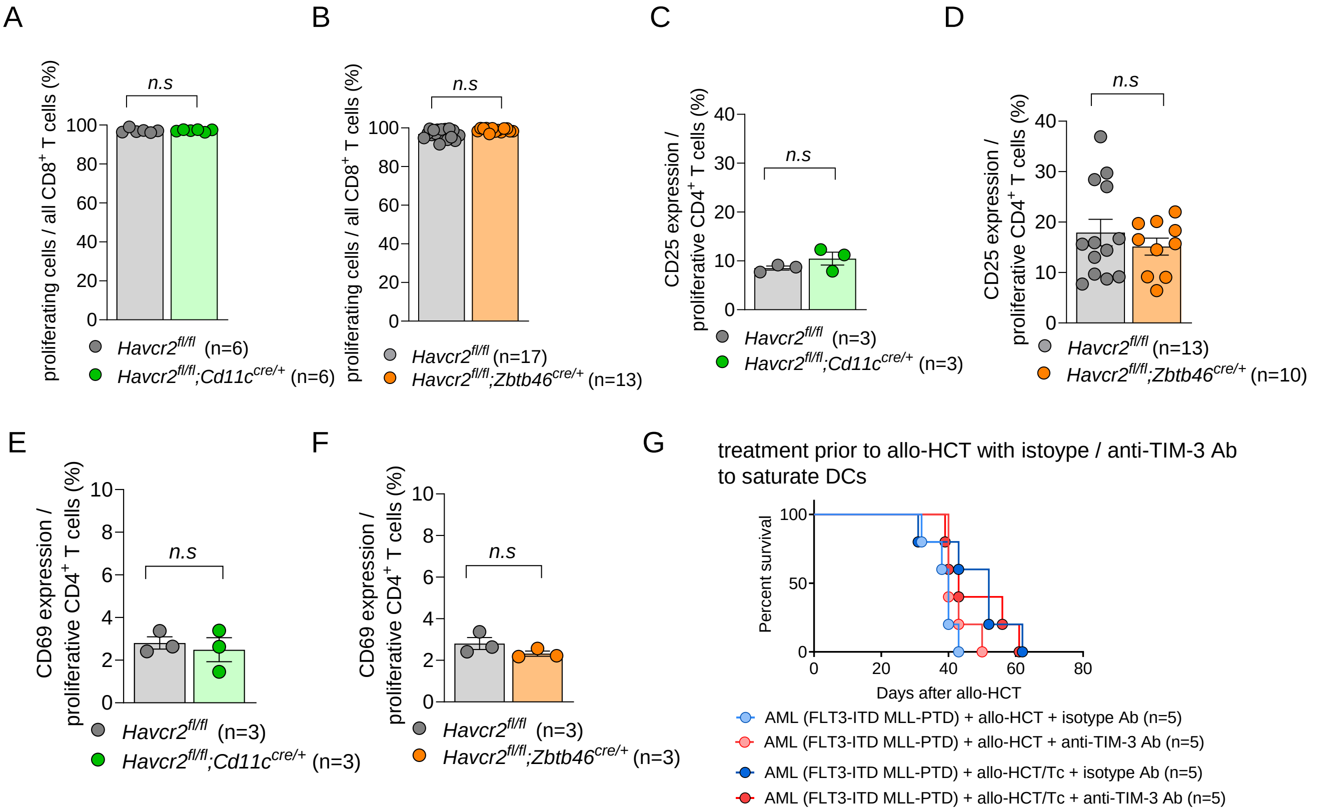

Suppl. Figure 7

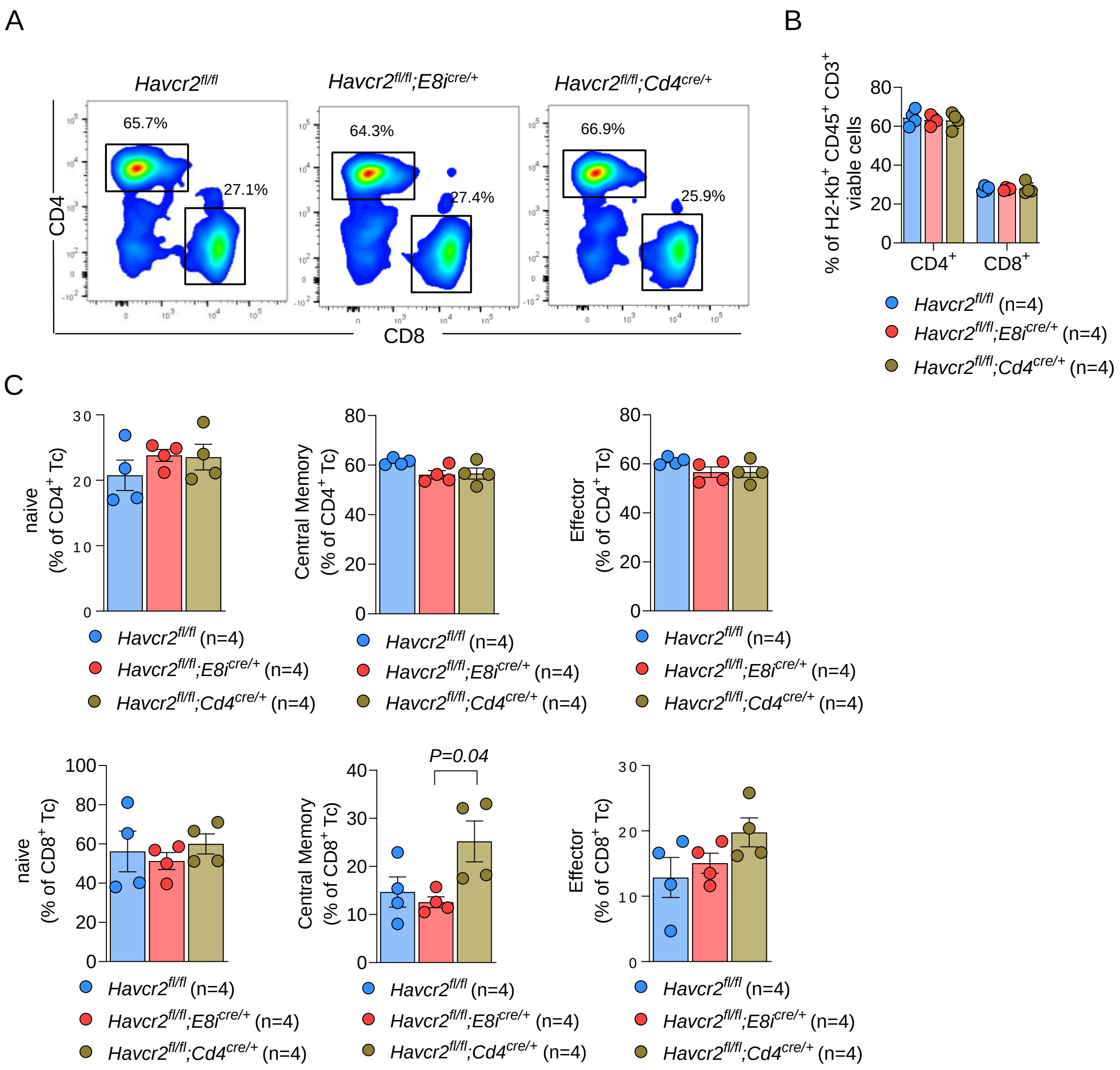

Suppl. Figure 8

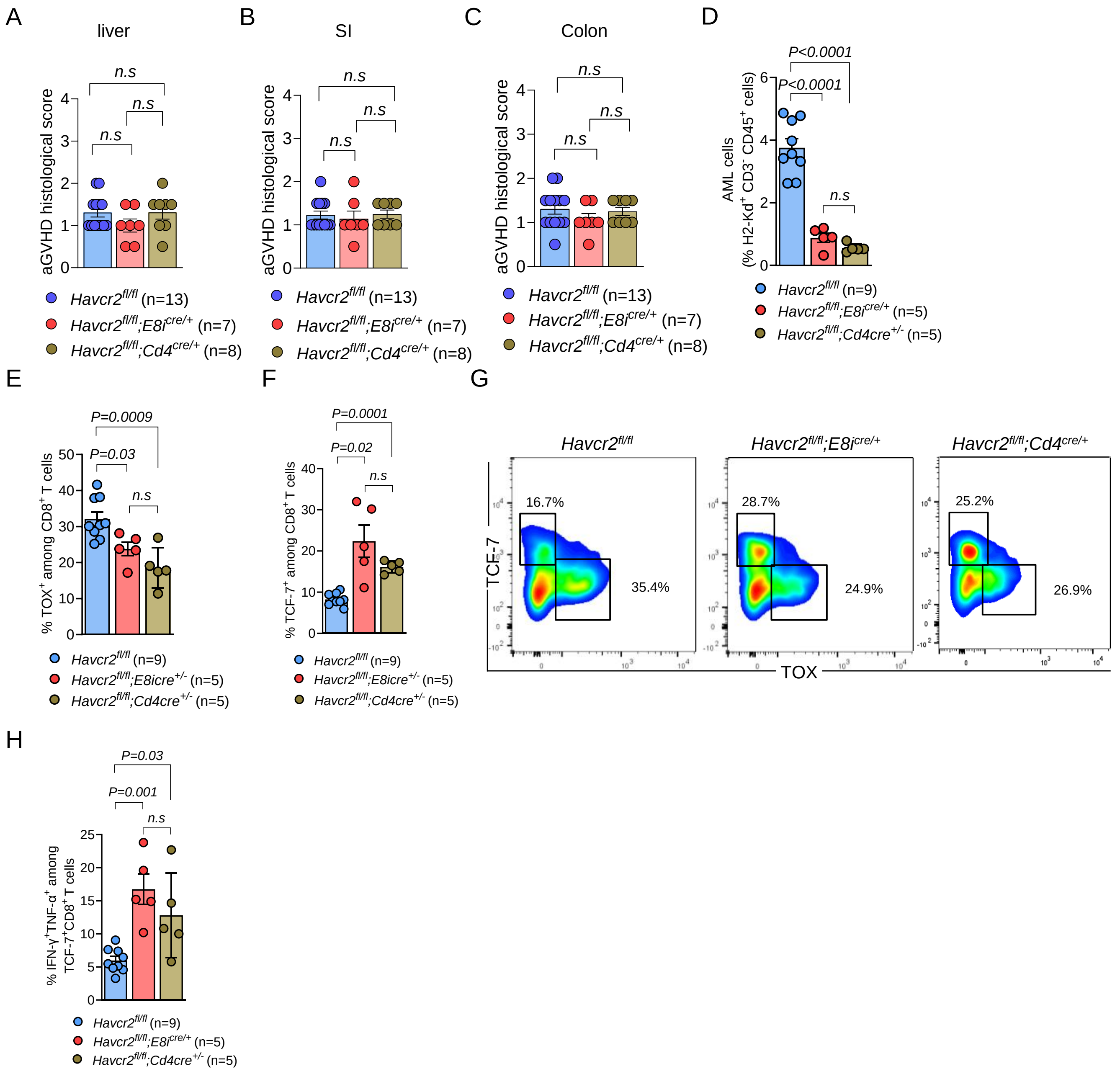

Suppl. Figure 9

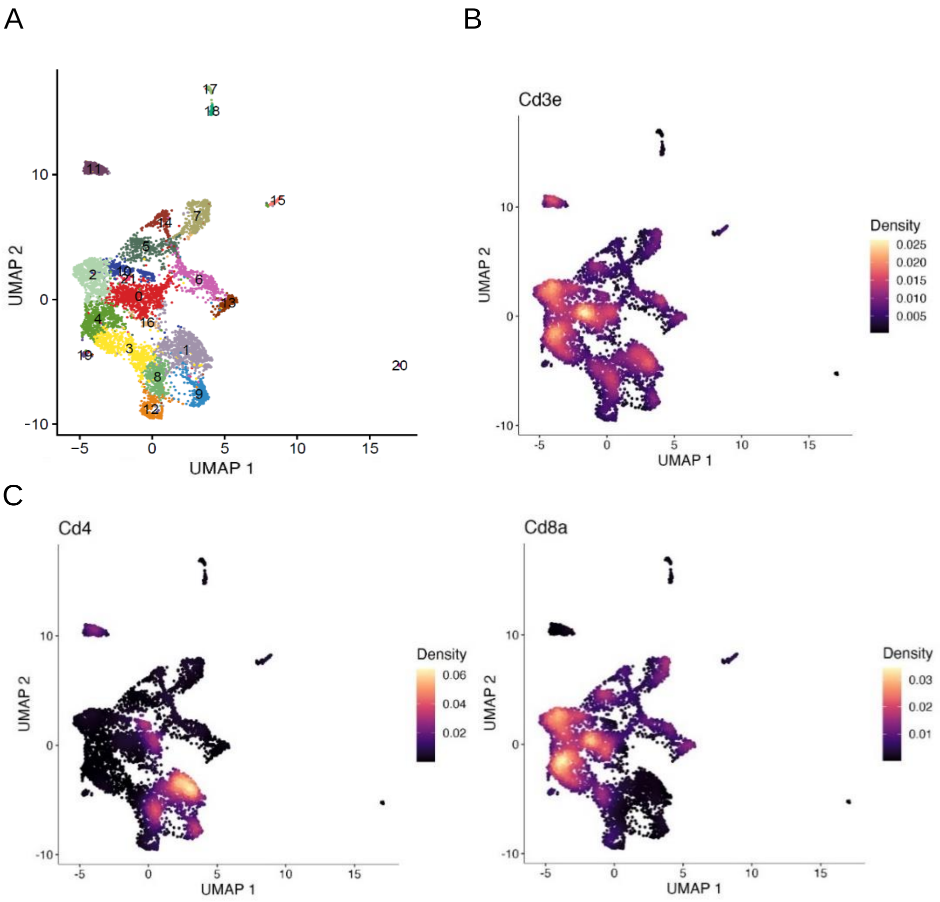

Suppl. Figure 10

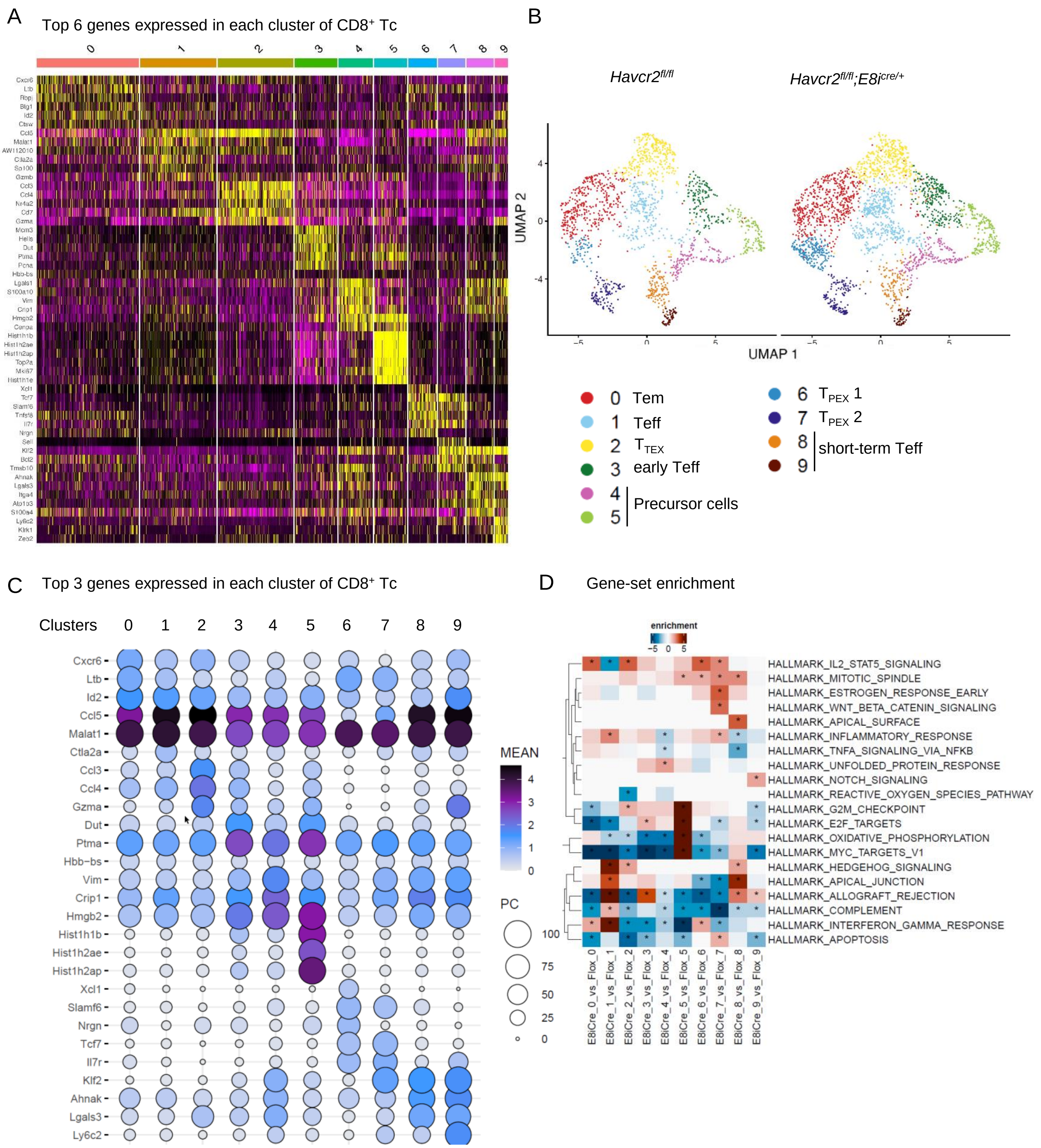

Suppl. Figure 11

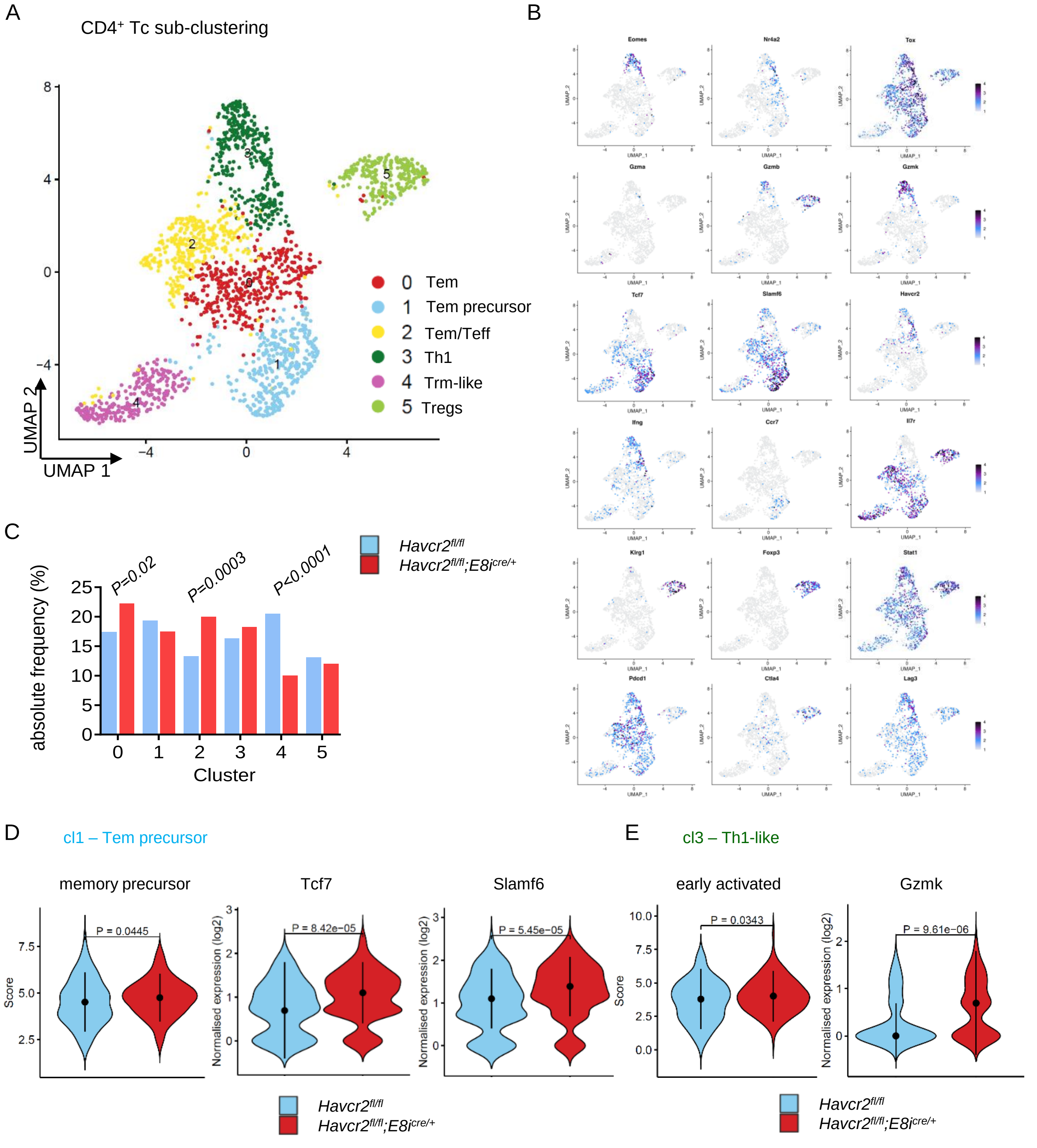

Suppl. Figure 12

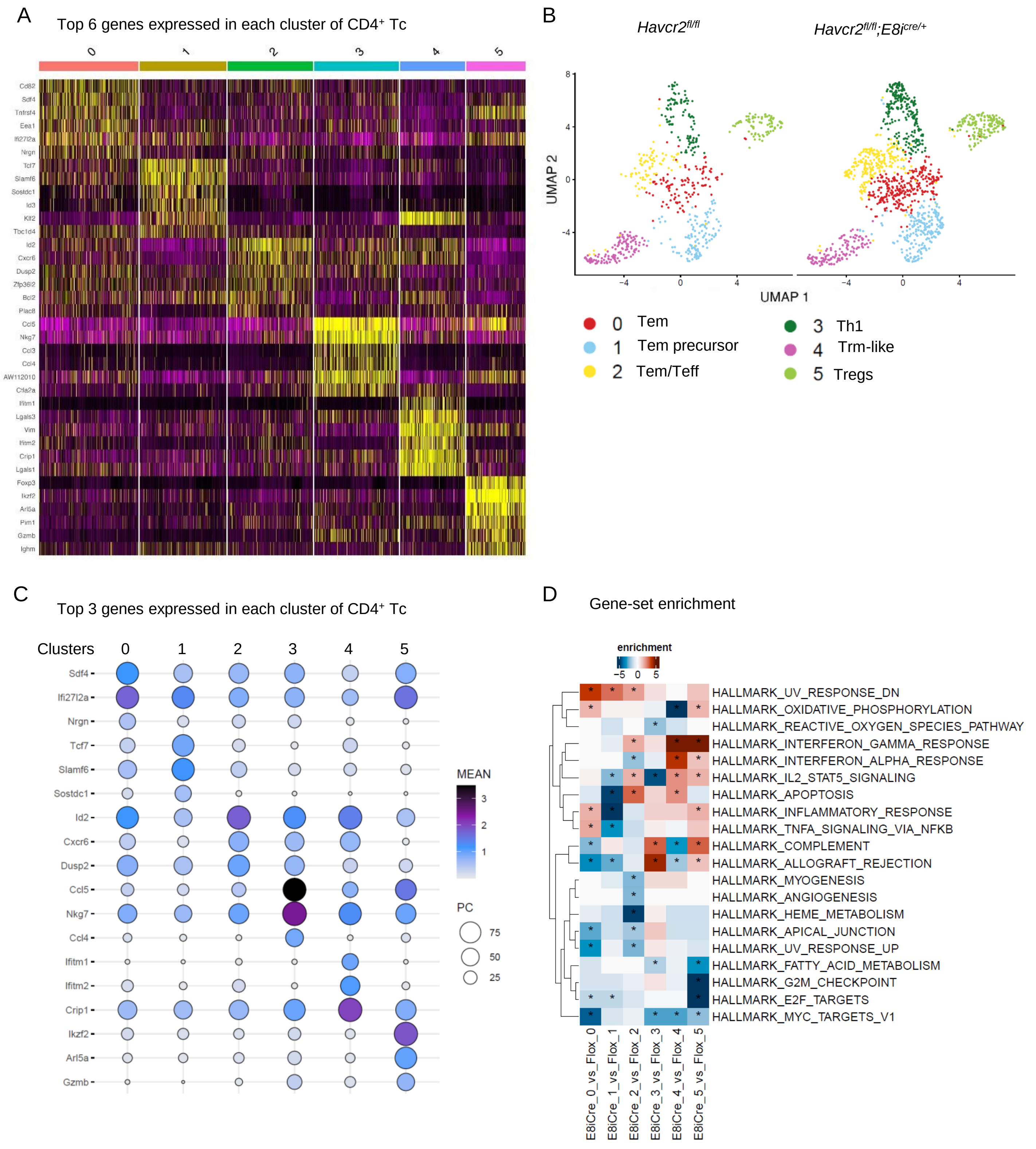

Suppl. Figure 13

A

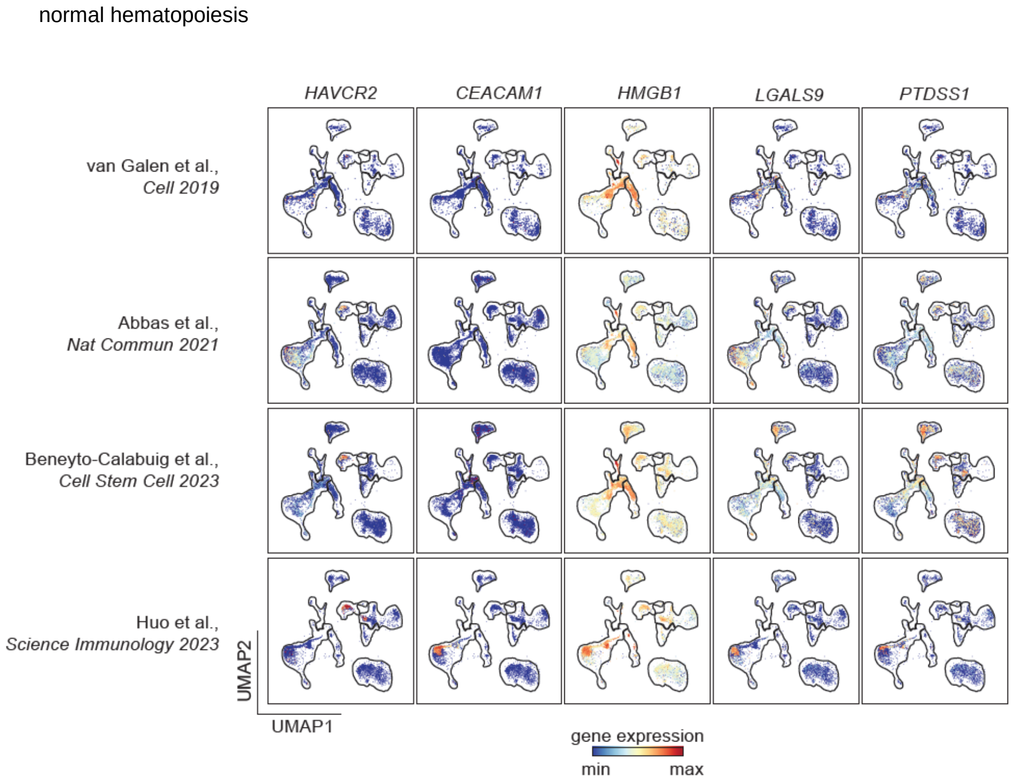

B

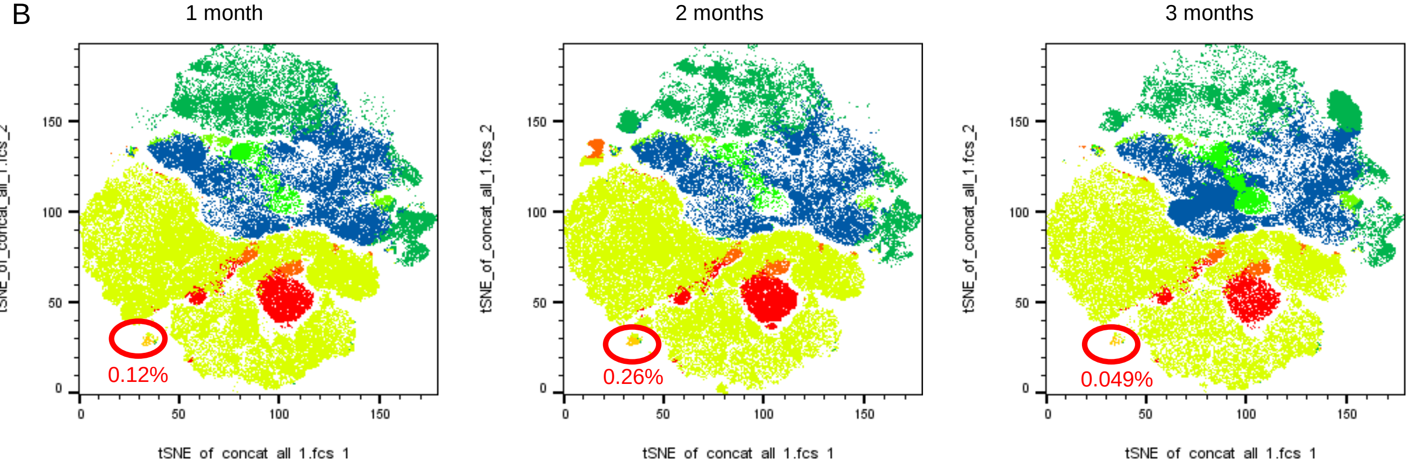

Suppl. Figure 14

A Peripheral blood

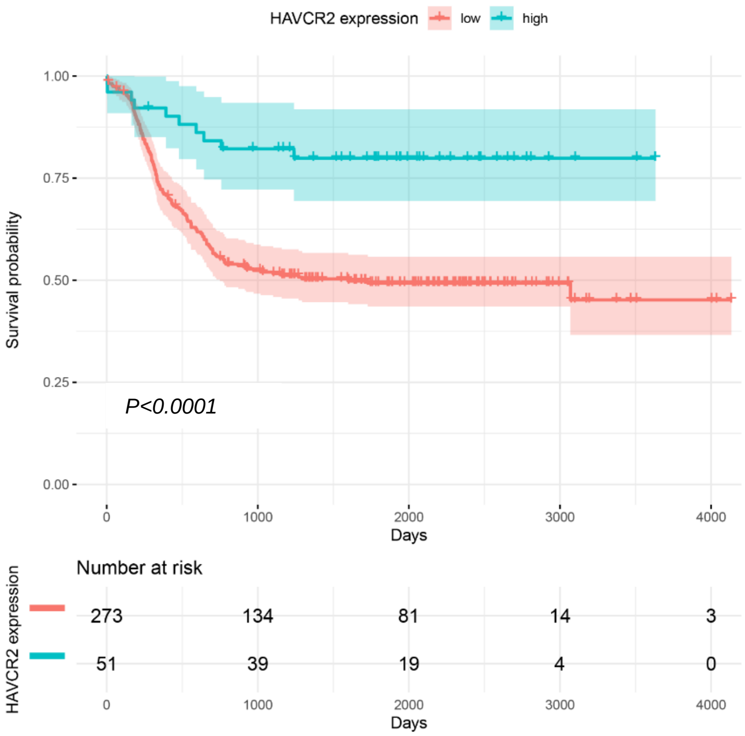

Supplementary material

Supplementary Figure legends

Suppl. Figure 1: TIM-3, Gal-9 and CAECAM-1 expression in T cells and AML cells

(A-B) C57BL/6 primary HSCs were transduced with the indicated oncogenes or gene fusions and RNA expression of **(A)** *Hmgb1* and **(B)** *Havcr2* was determined by qPCR. Gene expression data was normalized to *Hprt* expression and results show mean ± SEM from 3 independent experiments. Fold change was calculated in comparison to empty vector (dotted line). **(C)** Scatter plot showing Gal-9 production detected in supernatants of 32D cells after transduction with different oncogenes. Results show mean ± SEM from three independent experiments. P-values were calculated using Kruskal-Wallis One-Way ANOVA multiple comparisons test.

(D) TIM-3, Gal-9 and CEACAM-1 MFI expression on $CD8^+$ Tc isolated from untreated mice (n=5) or mice injected with FLT3-ITD MLL-PTD AML cells and undergoing allo-HCT/Tc at different time points after transplantation (n=5 for d10, d17 and d24) as indicated. Results represent mean ± SEM. The P-values were calculated using one-way Anova followed by Tukey's multiple comparisons test. **(E)** *Havcr2*, *Lgals9* and *Ceacam1* expression on leukemia cells isolated from untreated mice (n=5) or mice undergoing allo-HCT at different time points after transplantation (n=5 for d10, d17 and d24) as indicated. Gene expression data was normalized to *Hprt* expression and results represent mean ± SEM. Fold change was calculated in comparison to untreated mice. The P-values were calculated using one-way Anova followed by Tukey's multiple comparisons test.

Suppl. Figure 2: Anti-TIM-3 Ab treatment is not protective in the absence of allo-HCT

(A) Kaplan-Meier plot showing mouse survival in the indicated groups. Sublethally irradiated (total body irradiation (TBI) of 6Gy) C57BL/6 recipient mice were injected *i.v.* with FLT3-ITD MLL-PTD AML cells (C57BL/6 background). Mice were treated with either isotype Ab (mIgG1, n=10) or anti-TIM-3 Ab (clone 5D12, n=10) in the absence of allo-HCT. Results show 2 independent experiments and P-*v*alue was calculated using the 2-sided Mantel-Cox test.

Suppl. Figure 3: Anti-TIM-3 Ab treatment leads to reduced exhausted Tc subsets in leukemia-bearing mice

(A-C) BALB/c recipient mice were injected *i.v.* with WEHI-3B cells (BALB/c background) and $5x10^6$ allogeneic BM cells. Mice were injected with additional allogeneic Tc and treated with either isotype Ab (mIgG1, n=9) or anti-TIM-3 Ab (clone 5D12, n=9). The BM and spleens of these mice

were analyzed on day 23. **(A)** Representative FC plots displaying the proportions of exhausted TIM-3$^+$ PD-1$^+$ CD4$^+$ Tc and exhausted TIM-3$^+$ PD-1$^+$ CD8$^+$ Tc in the spleen of leukemia-bearing mice. **(B-C)** Relative TIM-3 MFI of TIM-3$^+$PD-1$^+$ **(B)** in CD4$^+$ Tc or **(C)** in CD8$^+$ Tc in the indicated organ was assessed by FC. Results represent mean ± SEM of 2 independent experiments and P-values were calculated using an unpaired Student's t-test. **(D-E)** The potential interference of anti-TIM-3 blockade with TIM-3 detection via FC was assessed on day 10 of *in vitro* Tc stimulation. Tc were then incubated with 10 µg/mL of anti-TIM-3 (clone 5D12) or isotype Ab (mIgG1) for 2 hours. **(D)** Overlaid histograms showing the expression of TIM-3 after the cells had been exposed to anti-TIM-3 Ab (red line) or the isotype Ab (dark dotted line). Exposure to anti-TIM-3 does not reduce TIM-3 detection compared to exposure to isotype Ab. Representative data from one of three biological replicates. **(E)** The bar diagram shows the relative TIM-3 MFI among viable cells. Results show mean ± SEM of 3 independent experiments.

Suppl. Figure 4: Metabolic changes in Tc isolated from anti-TIM-3 treated mice

(A, B) Gene-set-enrichment analysis (GSEA) using the R package "fgsea" with metabolic pathway definitions from KEGG and measured fold changes as input. First, metabolomics data was quantile normalized to compensate for cell size effects before calculating the logarithm of the fold change as a measure of difference between conditions. To compensate for method bias, only the sub-set of KEGG pathways that was covered by the employed metabolomics platform was considered. **(A)** The significance scores (P values) of the top 10 enriched pathways are displayed. **(B)** The enrichment plot for pathway "Glycoysis/Gluconeogenesis".

Suppl. Figure 5: Anti-TIM-3 Ab treatment after allo-HCT induces changes in myeloid populations

(A) Heatmap showing the median expression of the 27 indicated markers used to identify the different Tc subsets. **(B)** Scaled expression of 9 phenotypic or functional antigens with the use of the FlowSOM algorithm among 8 myeloid phenotypic subsets. Log fold change of isotype Ab treatment compared to anti-TIM-3 Ab treatment is shown (blue color depicts higher expression in isotype, red color higher expression in anti-TIM-3). Differentially expressed proteins with P-value < 0.05 tested with moderated t-test of limma are presented. All antibodies used for spectral FC are summarized in **Suppl. Table 3**.

Suppl. Figure 6: TIM-3 deletion in dendritic cells does not affect Tc proliferation and activation *in vitro*

(A-F) Tc labeled with CTV ("responders", BALB/c) were co-cultured for 6 days with allogeneic $CD3^{neg}$ cells ("stimulators", C57BL/6). Stimulator cells used were isolated from mice carrying TIM-3 deletion in all DCs (*$Havcr2^{fl/fl}$;$Cd11c^{cre+/-}$*) or in conventional DCs ($Havcr2^{fl/fl}$;$Zbrb46^{cre+/-}$) using the cre/lox system, as described previously (1). **(A)** The scatter plots show proliferating cells in all $CD8^+$ Tc (%) when Tc were exposed to *$Havcr2^{fl/fl}$* $CD3^-$ cells or *$Havcr2^{fl/fl}$;$Cd11c^{cre+/-}$* $CD3^-$ stimulator cells and **(B)** *$Havcr2^{fl/fl}$;$Zbrb46^{cre+/-}$* $CD3^-$ stimulator cells. **(C, D)** The scatter plots show $CD25^+$ cells among $CD4^+$ Tc (%) when Tc were exposed to *$Havcr2^{fl/fl}$* $CD3^-$ cells versus *$Havcr2^{fl/fl}$;$Cd11c^{cre+/-}$* $CD3^-$ cells **(C)** and *$Havcr2^{fl/fl}$* $CD3^-$ cells versus *$Havcr2^{fl/fl}$;$Zbrb46^{cre+/-}$* $CD3^-$ cells **(D)**. **(E, F)** The scatter plots show $CD69^+$ cells among proliferative $CD4^+$ Tc (%) when Tc were exposed to *$Havcr2^{fl/fl}$* $CD3^-$ cells versus *$Havcr2^{fl/fl}$;$Cd11c^{cre+/-}$* $CD3^-$ cells **(E)** and *$Havcr2^{fl/fl}$* $CD3^-$ cells versus *$Havcr2^{fl/fl}$;$Zbrb46^{cre+/-}$* $CD3^-$ cells **(F)**. The proportion of proliferative cells or expression of activation markers was quantified at day 6 as the percentage of CTV^{low} cells on responder Tc. **(G)** Kaplan-Meier plot showing mouse survival in the indicated groups. C57BL/6 recipient mice were injected i.v. with FLT3-ITD MLL-PTD AML cells (C57BL/6 background) and $5x10^6$ allogeneic BM. Mice were injected with additional allogeneic Tc when indicated. When indicated, mice were treated with either isotype Ab (mIgG1, n=5) or anti-TIM-3 Ab (clone 5D12, n=5) day -4 and day -1 prior to the allo-HCT. P-value was calculated using the 2-sided Mantel-Cox test.

Suppl. Figure 7: Tc subsets in the $Havcr2^{cko}$ mice

(A) Representative flow cytometry analysis of Tc subsets in the spleen of *$Havcr2^{fl/fl}$*, *$Havcr2^{fl/fl}$;$E8i^{cre/+}$* or *$Havcr2^{fl/fl}$;$Cd4^{cre/+}$* mice. **(B)** Percentage of $CD4^+$ and $CD8^+$ Tc subsets among $H2\text{-}Kb^+$ $CD45^+$ $CD3^+$ Tc in *$Havcr2^{fl/fl}$* (n=4), *$Havcr2^{fl/fl}$;$E8i^{cre/+}$* (n=4) or *$Havcr2^{fl/fl}$;$Cd4^{cre/+}$* (n=4) mice under steady state conditions. **(C)** Percentage of naïve, central memory and effector (based on $CD44^+$ / $CD62L^+$ cells) among $CD4^+$ and $CD8^+$ Tc subsets in *$Havcr2^{fl/fl}$* (n=4), *$Havcr2^{fl/fl}$;$E8i^{cre/+}$* (n=4) or *$Havcr2^{fl/fl}$;$Cd4^{cre/+}$* (n=4) mice under steady state conditions. P-values were calculated using a two-way Anova followed by Tukey's multiple comparisons test.

Suppl. Figure 8: Impact of anti-TIM-3 Ab treatment on aGVHD severity, tumor load and Tc phenotypes

(A-C) BALB/c recipient mice were injected *i.v.* with WEHI-3B AML cells (BALB/c background) and allogeneic *$Havcr2^{fl/fl}$* (n=13), *$Havcr2^{fl/fl}$;$E8i^{cre/+}$* (n=7) or allogeneic *$Havcr2^{fl/fl}$;$Cd4^{cre/+}$* (n=8) BM and Tc. Organs were collected at day 10-18 after allo-HCT and the scatter plots show the

histopathological aGVHD severity in the liver **(A),** SI **(B)** and the colon **(C)**. **(D-H)** The percentage of **(D)** AML cells (H2-Kd$^+$ CD3$^-$ CD45$^+$ cells) **(E)** TOX$^+$ among CD8$^+$ Tc **(F)** TCF-7$^+$ among CD8$^+$ Tc were assessed in the spleen. **(G)** Representative FC plots displaying the proportions of TOX$^+$ and TCF-7$^+$ among CD8$^+$ Tc. **(H)** Percentage of INF-γ$^+$ TNF-α$^+$ among TCF-7$^+$ CD8$^+$ Tc (T_{PEX} subset) after pan-Tc stimulation for 5 hours (Cell Stimulation Cocktail, eBiosciences). Results show mean ± SEM from 3 independent experiments. P-values were calculated using a two-way Anova followed by Tukey's multiple comparisons test.

Suppl. Figure 9: Sub-clustering of scRNAseq analysis

(A-C) BALB/c recipient mice were injected *i.v.* with WEHI-3B cell line (BALB/c background) and 5x10^6 allogeneic *Havcr2$^{fl/fl}$* or *Havcr2$^{fl/fl}$;E8i$^{cre/+}$* BM and Tc as indicated for each group. Tc were isolated at day 23 and stained with an oligo-tagged H-2Kb (donor) Ab allowing analysis of scRNA-seq libraries from 4 donor CD3$^+$ Tc samples (n=2 *Havcr2$^{fl/fl}$* and n=2 *Havcr2$^{fl/fl}$;E8i$^{cre/+}$*). **(A)** UMAP visualization revealed 21 distinct clusters before exclusion of non CD3$^+$ Tc. **(B)** Overall expression of the CD3e gene in each cluster **(C)**. The analysis was then divided into CD4$^+$ and CD8$^+$ Tc sub-clustering.

Suppl. Figure 10: Genetic TIM-3 deletion in CD8$^+$ Tc leads to remodeling of CD8$^+$ Tc subsets

(A-C) BALB/c recipient mice were injected *i.v.* with WEHI-3B cell line (BALB/c background) and 5x10^6 allogeneic *Havcr2$^{fl/fl}$* (n=2) and *Havcr2$^{fl/fl}$;E8i$^{cre/+}$* (n=2) BM and Tc. Tc were isolated at day 23 and stained with an oligo-tagged H-2Kb (donor) Ab allowing scRNA-seq analysis of selected donor Tc. **(A)** Heatmap showing the Top 6 marker genes expressed for each clusters of CD8$^+$ Tc. **(B)** UMAP showing metaclustering of CD8$^+$ Tc in AML-bearing mice transferred with *Havcr2$^{fl/fl}$* (left panel) and *Havcr2$^{fl/fl}$;E8i$^{cre/+}$* (right panel) BM/Tc. (Tem: effector memory Tc; Teff: effector Tc). **(C)** Dot plot showing the Top 3 marker genes expressed for each cluster of CD8$^+$ Tc. **(D)** The gene-set analysis represents the Top 20 most significantly regulated Hallmark gene sets in each individual CD8$^+$ Tc cluster.

Suppl. Figure 11: Deletion of TIM-3 in CD8$^+$ Tc leads to changes in CD4$^+$ Tc responses

(A-E) BALB/c recipient mice were injected i.v. with WEHI-3B cell line (BALB/c background) and 5x10^6 allogeneic *Havcr2$^{fl/fl}$* and *Havcr2$^{fl/fl}$;E8i$^{cre/+}$* BM and Tc. Tc were isolated at day 23 and stained with H-2Kb (donor) Ab allowing analysis of scRNA-seq libraries from 4 donor CD3$^+$ Tc samples (n=2 *Havcr2$^{fl/fl}$* and n=2 *Havcr2$^{fl/fl}$;E8i$^{cre/+}$*). **(A)** The UMAP visualization shows 6 clusters of CD4$^+$ Tc isolated from AML-bearing mice. **(B)** Feature plots showing the expression level of different marker genes expression relevant for the characterization of the clusters. **(C)** Bar diagram

representing the frequency of the different CD4$^+$ Tc clusters. Adjusted P-values were calculated using Fisher's test. **(D-E)** Scores and expression levels of functional signature and key genes differentially expressed in AML-bearing mice receiving *Havcr2*$^{fl/fl}$ or *Havcr2*$^{fl/fl}$;*E8i*$^{cre/+}$ Tc in (**D**) cluster 1 or in (**E**) cluster 3. (Tem: effector memory Tc; Teff: effector Tc; Trm-like: resident memory-like Tc).

Suppl. Figure 12: Genetic TIM-3 deletion in CD8$^+$ Tc leads to remodeling of Tc subsets

(A-C) BALB/c recipient mice were injected *i.v.* with WEHI-3B cell line (BALB/c background) and 5x10^6 allogeneic *Havcr2*$^{fl/fl}$ (n=2) and *Havcr2*$^{fl/fl}$;*E8i*$^{cre/+}$ (n=2) BM and Tc. Tc were isolated at day 23 and stained with H-2Kb (donor) Ab allowing scRNA-seq analysis of selected donor Tc. **(A)** Heatmap showing the Top 6 marker genes expressed for each cluster of CD4$^+$ Tc. **(B)** UMAP showing metaclustering of CD4$^+$ Tc in AML-bearing mice transferred with *Havcr2*$^{fl/fl}$ (left panel) and *Havcr2*$^{fl/fl}$;*E8i*$^{cre/+}$ (right panel) BM/Tc. **(C)** Dot plot showing the Top 3 marker genes expressed for each cluster of CD4$^+$ Tc. **(D)** The analysis of enriched gene sets represents the Top 20 most significantly regulated terms from Hallmark gene set collection in each individual CD4$^+$ Tc cluster.

Suppl. Figure 13: Expression of TIM-3 on Tc in patients after transplant

(A) Scaled gene expression of *HAVCR2*, *CEACAM1*, *HMGB1*, *LGALS9* and *PTDSS1* across healthy donor BM scRNA-seq profiles. **(B)** tSNE representation of concatenated data from available patients 1 month (left panel), 2 months (middle panel) and 3 months (right panel) after allo-HCT.

Suppl. Figure 14: TIM-3 gene expression in PBMCs of AML patients correlates with OS

(A) Probability of survival stratified according to high versus low *HAVCR2* gene expression in PB. RNAseq data was collected from the GenomicDataCommons (GDC) library. Gene expression more than one standard deviation above the mean was considered as high expression.

Supplementary tables

Supplementary Table 1: Cell Ranger raw metrics summary

SAMPLE	E8iCre_1	E8iCre_2	Flox_1	Flox_2
Estimated.Number.of.Cells	4,328	3,208	1,511	6,737
Mean.Reads.per.Cell	35,430	53,166	80,183	27,728
Median.Genes.per.Cell	1,741	1,979	2,093	1,322
Number.of.Reads	153,339,249	170,554,972	121,156,528	186,805,706
Valid.Barcodes	97.8%	97.8%	97.8%	97.8%
Sequencing.Saturation	52.9%	62.4%	71.6%	45.5%
Q30.Bases.in.Barcode	95.1%	95.1%	95.0%	95.2%
Q30.Bases.in.RNA.Read	91.3%	90.4%	90.4%	91.1%
Q30.Bases.in.UMI	94.7%	94.5%	94.3%	94.6%
Reads.Mapped.to.Genome	94.6%	95.4%	95.1%	94.2%
Reads.Mapped.Confidently.to.Genome	88.8%	84.6%	85.7%	88.5%
Reads.Mapped.Confidently.to.Intergenic.Regions	4.6%	4.4%	4.4%	4.3%
Reads.Mapped.Confidently.to.Intronic.Regions	22.6%	21.9%	18.8%	17.7%
Reads.Mapped.Confidently.to.Exonic.Regions	61.6%	58.3%	62.5%	66.5%
Reads.Mapped.Confidently.to.Transcriptome	58.6%	54.7%	59.2%	63.4%
Reads.Mapped.Antisense.to.Gene	1.5%	1.8%	1.7%	1.5%
Fraction.Reads.in.Cells	93.1%	92.2%	89.4%	87.1%
Total.Genes.Detected	18,686	18,562	17,432	18,373
Median.UMI.Counts.per.Cell	4,299	5,384	6,642	3,219

Supplementary Table 2: Patient characteristics

				Patients with AML (n = 22)
**Patient Characteristics**				
	Age		years, median (range)	70 (21 - 75)
	Gender	female	no. (%)	8(36)
		male		14 (64)
**Disease Characteristics**				
	AML Type	de novo AML	no. (%)	11 (50)
		AML-MR		4 (18)
		t-AML		3 (14)
		s-AML (MPN)		3 (14)
		AML + myeloid sarcoma		1 (5)
	Mutation Status			
		NPM1	mutated, no. (%)	4 (18)
		CBFB-MYH11	mutated, no. (%)	3(14)
		TP53	mutated, no. (%)	4(18)
		FLT3 ITD	mutated, no. (%)	5(23)
**Induction Therapy**				
	Chemotherapy		no. (%)	5 (23)
	Chemotherapy + GO			4 (18)
	HMA + Venetoclax			11 (50)
	Chemotherapy + TKI			5 (11)23

**Transplant-related Characteristics**			
Number of allo-HCT	1		19 (86)
	2		3 (14)
Remission before allo-HCT			
	CR	no. (% transplanted pat.)	18 (82)
	Molecular persistence		4 (18)
Conditioning Regimen			
	RIC	no. (% transplanted pat.)	22 (100)
Donor Type			
	MRD	no. (% transplanted pat.)	22(100)
Graft Source			
	PBSC	no. (% transplanted pat.)	20 (91)
	BM		2(9)
**Outcome**			
Remission after allo-HCT	CR	no. (% transplanted pat.)	21 (95)
	Molecular persistence		1 (5)
Survival after allo-HCT		days, mean (range)	562 (89 - 854)
Cause of death			
	leukemia	no. (%)	9 (16)
	GvHD		0 (0)
	infection		1 (3)

Human PB and/or BM samples were derived from AML patients treated at the Department of Medical Oncology, Dana-Farber Cancer Institute, Boston, Massachusetts, USA, after prior informed consent (n=22). GO: gemtuzumab ozagamicin. HMA: hypomethylating agents (decitabine, azacytidine). TKI: tyrosine kinase inhibitors (midostaurin, gilteritinib). CR: complete remission. BP: blast persistence. MAC: myeloablative conditioning. RIC: reduced intensity conditioning. MRD: HLA-matched related donor. MUD: HLA-matched unrelated donor. MMUD: HLA-mismatched unrelated donor. PBSC: peripheral blood-derived hematopoietic stem cell graft for allo-HCT.

Supplementary Table 3: List of antibodies used for spectral flow cytometry

Antigen	Fluorochrome	Clone	Manufacturer
**CD8a**	BUV 805	53-6.7	BD Biosciences
**CD11b**	BUV737	M1/70	BD Biosciences
**H-2kd**	BUV615	SF1-1.1	BD Biosciences
**CD19**	BUV 661	1D3	BD Biosciences
**Ly6G**	BUV563	1A8	BD Biosciences
**H-2Kb**	BUV496	AF6-88.5	BD Biosciences
**CD172a (Sirpα)**	BUV395	P84	BD Biosciences
**XCR1**	BV785	ZET	BioLegend
**CD88**	BV750	20/70	BD Biosciences
**Ly6C**	BV 711	HK1.4	BioLegend

**CD45**	BV 650	30-F11	BioLegend
**CX3CR1**	BV 605	SA011F11	BioLegend
**CD90.2**	BV 570	30-H12	BioLegend
**Ki67**	BV480	B56	BD Biosciences
**CD45R (B220)**	Pac Blue	RA3-6B2	BD Biosciences
**Ox40**	BV 421	Ox-86	BioLegend
**Siglec-H**	PerCP-eFluor710	551	eBioscience
**CD103**	Biotin	2E7	BioLegend
**CD4**	Spark Blue 550	GK1.5	BioLegend
**TIM-3**	PE-Fire 810	RMT3-23	BioLegend
**CD11c**	PE-Cy5.5	N418	eBioscience
**MHCII**	PE-Cy5	M5/114.15.2	BioLegend
**F4/80**	PE-Dazzle 594	BM8	BioLegend
**IL4Ra**	PE	hIL4R-M57	BioLegend
**CD38**	APC-Fire 810	90	BioLegend
**GR1**	Alexa Fluor 700	RB6-85C	BioLegend
**Galectin-9**	APC	108A2	BioLegend
**CD44**	BUV 737	IM7	BD Biosciences
**GITR**	BUV563	DTA-1	BD Biosciences
**CD69**	BUV395	H1.2F3	BD Biosciences
**CD45**	BV 785	30-F11	BioLegend
**ICOS**	BV750	C398.4A	BioLegend
**NK1.1**	BV711	PK136	BioLegend
**CD226**	BV 650	TX42.1	BioLegend
**CTLA4 (CD152)**	BV 605	UC10-4B9	BioLegend
**CD62L**	BV 570	MEL-14	BioLegend
**Granzyme B**	Pacific blue	GB11	BioLegend
**KLRG1**	BV 421	2F1/KLRG1	BioLegend
**CD39**	PerCP-eFlour710	24DMS1	eBioscience
**CD73**	BB660	TY/23	BD Biosciences
**TCF1**	AF488	C63D9	Cell Signaling Technologies
**CD279**	PE-Fire 810	29F.1A12	BioLegend
**T-Bet**	PE-Cy7	4B10	BioLegend
**Foxp3**	PE-Cy5.5	FJK-16s	eBioscience
**TCRbeta**	PE-Cy5	H57-597	BioLegend
**TIGIT**	PE-Dazzle594	1G9	BioLegend
**TOX**	PE	REA473	Miltenyi
**TIM-3**	APC	RMT3-23	BioLegend

Supplementary Table 4: List of Abs used for flow cytometry

Antigen	Fluorochrome	Clone	Manufacturer
**H-2kd**	PerCP Cy5.5	SF1-1.1	BioLegend
**H-2kb**	APC	AF6-88.5.5.3	eBioscience
**CD45.1**	FITC	A20	BioLegend
**CD45.2**	Pacific Blue	104	BioLegend
**CD3**	BV711	17A2	BioLegend
**CD4**	PE-Cy7	GK1.5	BioLegend
**CD8a**	BV605	53-6.7	BioLegend
**CD62L**	BV510	MEL-14	BioLegend
**CD44**	PE	IM7	BioLegend
**CD25**	PE	PC61	BioLegend
**CD45**	PE Cy7	30-F11	BioLegend
**CD69**	BV605	H1.2F3	BioLegend
**TIM-3**	BV785	RMT3-23	BioLegend
**TIM-3**	BV650	7D3	BD Biosciences
**PD-1**	PE Cy7	29.F1A.12	BioLegend
**CD11b**	BV510	M1/70	BioLegend
**CD117**	AF647	2B8	BD Biosciences
**Gal-9**	APC	108A2	BD Biosciences
**Gal-9**	FITC	9M1-3	BioLegend
**Ceacam-1**	APC	ASL-32	BioLegend
**Ceacam-1**	PE Cy7	MAb-CC1	BioLegend
**Hmgb-1**	PE	3E8	BioLegend
**TCF-1/7**	AF488	S33-966	BD Biosciences
**TOX**	PE	REA473	Miltenyi biotec
**IFN-γ**	BV711	XMG1.2	BioLegend
**TNF-α**	Pacific Blue	MP6-XT22	BioLegend
**CD11c**	PE	N418	BioLegend

Supplementary Methods

GVL mouse models

GVL experiments were performed as previously described (2, 3). Briefly, recipients were lethally irradiated using a ^{137}Cs source. Total body irradiation dose was split into two equal doses at an interval of at least 4 hours to minimize toxicity. On the same day, mice were injected *i.v.* with leukemia cells and donor BM cells (when indicated). At day 2, spleens of healthy donors animals were enriched for CD3$^+$ Tc by negative selection using Pan T-cell Isolation Kit II (Miltenyi Biotech, USA) and the MACS cell separation system (Miltenyi Biotec) according to the manufacturer's instructions. Tc purity (>90%) was confirmed by flow cytometry. CD3$^+$ Tc were injected (*i.v.*) to the recipient mice. When indicated, mice were treated with anti-mouse anti-TIM-3 (5D12) / isotype Ab (mIgG1) or human anti-TIM-3 sabatolimab (Novartis) / vehicle (Glucose solution 5%) from day 7 to day 28 with a 3-day interval. When indicated, donor BM cells and Tc were isolated from Havcr2^{cko} mice.

WEHI-3B leukemia model

BALB/c recipients were transplanted with 10x10^3 WEHI-3B AML cells and 5x10^6 allogeneic C57/BL6 BM cells *i.v.* after lethal irradiation with two doses of 5.08 Gy TBI. On day 2, 2x10^5 C57/BL6 allogeneic splenic CD3$^+$ Tc were injected (*i.v.*) following initial transplantation.

FLT3-ITD MLL-PTD AML leukemia model

C57BL/6 recipients were transplanted with 5x10^3 FLT3-ITD MLL-PTD AML cells and 5x10^6 BALB/c BM cells *i.v.* after lethal irradiation with two doses of 6 Gy TBI. On day 2, 3x10^5 BALB/c allogeneic splenic CD3$^+$ Tc were injected (*i.v.*) as previously reported (4, 5). For the leukemia model in absence of allo-HCT, C57BL/6 recipients were transplanted with 2x10^6 FLT3-ITD MLL-PTD AML cells *i.v.* after sublethal irradiation of 6 Gy TBI.

MOLM-13^{luc+} FLT3-ITD AML xenograft model

Immunodeficient *Rag2$^{–/–}$Il2rg$^{–/–}$* recipients were irradiated with 3.5 Gy (sublethal irradiation) prior to intravenous injection of 1x10^5 MOLM-13^{luc+} cells. On day 2, 5x10^4 human CD3$^+$ T-cells were injected (*i.v.*).

Human primary AML xenograft model

Rag2$^{–/–}$Il2rg$^{–/–}$ recipients were irradiated with 3.5 Gy (sublethal irradiation) prior to intravenous injection of 10x10^6 CD3-depleted human primary AML cells. Human primary AML cells were used

fresh and were isolated by FICOLL density centrifugation. $CD3^+$ Tc depletion was performed by magnetic separation. On day 2, $5x10^4$ human $CD3^+$ Tc isolated from peripheral blood of healthy donors were injected *i.v*.

GVHD mouse model

Briefly, BALB/c recipient mice were irradiated (using a lethal dose divided into two doses of 5.08Gy within a 4-hour interval) prior to the injection (*i.v.*) of allogeneic $5x10^6$ C57BL/6 BM cells and $4x10^5$ Tc. From day 1 to day 5, mice were injected (*i.p.*) with 150µg of anti-TIM-3 / isotype, anti-PD-1 (RMP1-14) / isotype (anti-rat IgG2a) or anti-CTLA-4 (9D9) / isotype (mIgG2b) Ab. At d7, mice were sacrificed by cervical dislocation. Organs (liver, SI and colon) were collected and stained with hematoxylin and eosin. Histopathological GVHD severity was assessed on the basis of a published histopathology scoring system (6) by an experienced pathologist blinded to the experimental groups.

Generation of retroviral supernatants

For all transductions, we used the Murine Stem Cell Virus (MSCV) retroviral expression vector system. We used the MSCV backbone plasmid with different oncogenes and IRES-GFP (pMIG). For generation of virus supernatant, we used Platinum-E (Plat-E) retroviral packaging cell line. Plat-E cells were transfected with 10 µg MSCV vectors using Lipofectamin™2000 transfection reagent (Invitrogen™) according to manufacturer's protocol. Transfection was checked using fluorescence microscopy after 24h and transfection mix was replaced by 5 ml of fresh Dulbecco's Modified Eagle Medium (DMEM, Gibco) supplemented with 10% FCS and 1% P/S. Virus supernatant was collected three subsequent times with 12h between. Virus supernatant was filtered using a 0.45µm syringe filter and stored for less than one week at 4°C.

Viral transduction of murine primary HSCs cells with oncogenic driver mutations or gene fusions

To generate empty vector (EV)-tg, FLT3-ITD-tg, KRAS-G12V-tg, cKIT-D816V-tg, JAK2-V617F-tg, FIP1L1-PDGFRα-tg, NRAS-G12D-tg, cMYC-tg and CSFR3-T618L-tg HSCs cells, BALB/c or C57BL/6 mice were injected with 100 mg/kg 5-fluorouracil (Medac GmbH). 4 days after 5-fluorouracil treatment, primary bone marrow cells (HSCs) were isolated and stimulated overnight with growth factors (mIL-3 10 ng/mL, mIL-6 10 ng/mL and mStem Cell Factor 14.3 ng/mL, all from Peprotech) as described previously (2, 7). Cell were transduced in DMEM medium supplemented with 10% FCS, 1% P/S, growth factors mentioned above and polybrene (Sigma-Aldrich, 4 µg/mL). Retroviral supernatant was added to the medium before and 3 rounds of spin infection (each 12

hours) were performed (1200g, 90 min, 32°C). Transduction efficiency was measured using flow cytometry detecting GFP-expressing cells.

In vitro Tc proliferation assays

αCD3/CD28-mediated Tc stimulation experiments were performed using splenic mouse Tc purified using the pan Tc isolation kit (Miltenyi Biotec) cocultured with Dynabeads™ Mouse T-Activator CD3/CD28 (ratio 1:1, Thermofisher Scientific) and mIL-2 (30U/mL). When indicated, anti-TIM-3 or isotype Ab was added to the culture (10 µg/mL, every two days). For *in vitro* exhaustion analysis, cells were continuously exposed to CD3/CD28 Abs in the presence of mouse IL-2 for 14 days before analysis by FC.

Mixed Lymphocyte Reaction (MLR) were performed using CellTrace Violet (CTV)-labeled $CD3^+$ Tc (responders, $2x10^6$/mL) incubated with allogeneic $CD3^{neg}$ cells (stimulators, $1x10^6$/mL) in 96-well flat-bottom plates. Responder cells were isolated from $Havcr2^{cko}$ mice and CTV labeling (5 µM) was performed using CellTrace Violet™ Cell Proliferation Kit (ThermoFisher Scientific) according to the manufacturer's instructions. Cells were harvested on day 6 and Tc proliferation/activation was analyzed by FC.

In vitro killing assay

Splenic $Havcr2^{cko}$ mouse Tc were isolated using the pan Tc isolation kit (Miltenyi Biotec) and cultured with Dynabeads™ Mouse T-Activator CD3/CD28 (ratio 1:1, Thermofisher Scientific) and mIL-2 (30U/mL). Cells were split when needed and mIL-2 (30U/mL) was added to the culture every two days. On day 6, αCD3/CD28 beads were removed and Tc were cocultured with $WEHI\text{-}3B^{GFP+}$ cells for 6 hours at the indicated ratios. Specific killing of leukemia cells was analyzed by FC.

In vivo Bioluminescence imaging (BLI)

Luciferin (D-luciferin, potassium salt (S)-4,5-Dihydro-2-(6-hydroxy-2-benzothiazolyl)-4-thiazolecarboxylic acid potassium salt; Biosynth) was dissolved in H_2O and injected (*i.p.*, 150 µg/g body weight) 10 minutes before BLI. The mice were exposed to isoflurane anesthesia and imaged using an IVIS Lumina III *in vivo* imaging system (PerkinElmer) with an exposure time of 1 minute. The luciferase signal was quantified in photons per second per mouse. Acquisition, analysis, and visualization of BLI were performed using Living Image Software (PerkinElmer).

Liquid chromatography–mass spectrometry (LC–MS) metabolomic analysis

C57BL/6 recipients were transplanted with $5x10^3$ FLT3-ITD MLL-PTD AML cells and $5x10^6$ BALB/c BM cells *i.v.* after TBI. On day 2 post allo-HCT, $3x10^5$ BALB/c allogeneic splenic $CD3^+$ T-cells were

injected (*i.v.*). Mice were treated with 150µg of isotype (mIgG1) or anti-TIM-3 (clone 5D12) antibody starting from day 7 and every 3 days. On day 23, Tc were isolated from the spleen using pan T-cell isolation kit (Miltenyi biotec). Polar metabolites were extracted from $3x10^6$ cells per sample with 150µL of metabolite extraction solution (50% methanol, 30% acetonitrile, 20% water). Non-targeted metabolomics was carried out using an Agilent 1290 Infinity II UHPLC inline with a Bruker Impact II QTOF-MS operating in negative ion mode. Scan range was from 20 to 1050 Da. Mass calibration was performed at the beginning of each run. LC separation was on a Waters Atlantis Premier BEH ZHILIC column (100 x 2.1 mm, 1.7 µm particles), buffer A was 20 mM ammonium carbonate and 5 µM medronic acid in milliQ H_2O and buffer B was 90:10 acetonitrile:buffer A. The solvent gradient was from 95% to 55% buffer B over 14 minutes, flow rate was 180 µL/min, column temperature was 35 °C, autosampler temperature was 5 °C and injection volume was 3 µL. Data pre-processing including detection, deconvolution, and identification of features was carried out using Metaboscape version 2023 (Bruker). Only features with an average intensity in cell extracts of more than 1.5 fold the average intensity in blank samples were included in subsequent analyses. Quantile normalization of signal intensity and generation of volcano plot were performed in R version 4.2.1.

Single-cell energetic metabolism by profiling translation inhibition (SCENITH)

To assess the metabolic activity of Tc subsets, we used the method named single cell energetic metabolism by profiling translation inhibition (SCENITH) (8). Briefly, cell suspension was prepared from the spleens and isolated cells were divided into four different conditions. 1×10^6/mL splenocytes were plated for each conditions in RPMI medium supplemented with 10% FBS, 1% penicillin / streptomycin for 1 hour. Then, cells were treated for 30 minutes with Control (DMSO), 2-Deoxy-Glucose (2-DG; 100mM), Oligomycin (O; 1 µM), or a combination of 2DG and Oligomycin (DGO). Following metabolic inhibitors, O-propargyl-puromycin (OPP from the Click-iT™ Plus OPP Protein Synthesis Assay Kit, Invitrogen) was added to cultures for 30 minutes. After OPP treatment, cells were washed in cold PBS and stained with surface marker Abs for 30 min at 4 °C. Intracellular staining of OPP was performed as per manufacturer instructions. Finally, data acquisition was performed using a Fortessa flow cytometer. Calculation of the glycolytic capacity of $CD4^+$ and $CD8^+$ T cell subsets was calculated using the MFI of incorporated OPP in the corresponding Tc subsets.

Single-cell RNA sequencing (scRNA-Seq)

BALB/c recipients (n=2 per group) were transplanted with $5x10^3$ WEHI-3B AML cells and $5x10^6$ allogeneic C57BL/6 Havcr2^{cko} BM cells (*Havcr2$^{fl/fl}$* and *Havcr2$^{fl/fl}$;E8i$^{cre/+}$*) *i.v.* after TBI. On day 2,

2x10^5 C57/BL6 allogeneic Havcr2^{cko} splenic CD3$^+$ Tc were injected (*i.v.*). On day 23, the spleens were harvested and Tc were isolated using the pan-Tc isolation kit (Miltenyi Biotec). Cells were labelled with an oligo-tagged H-2Kb Ab (TotalSeq™-B0952, Biolegend) before performing cell count in 0.04 % BSA/PBS according to the 10x Genomics protocol. 10 000 CD3$^+$ cells were encapsulated into droplets with barcoded Gel Beads using the Chromium Controller Single-Cell Instrument (10X Genomics). Libraries were prepared using Chromium Next GEM Single Cell 3′ Reagent Kits v3.1 (10x Genomics). Generated scRNA Seq libraries were pooled and sequenced on a NOVASEQ6000 (Illumina). Paired-end reads were processed with 10x Genomics Cell Ranger 6.1.2 (9) using the Mouse mm10 (2020-A) transcriptome index from 10x Genomics. Summary quality metrics are provided in **Suppl. Table 1**. Downstream analysis was performed with the Seurat R package pipeline (V4) (10). The following quality filters have been applied: 5000 > nFeature_RNA > 200, percent.mt < 10. The 4 samples were integrated into a single dataset using the SCTransform based normalization using the following parameters: method = "glmGamPoi", vst.flavor = "v2", vars.to.regress = c("nCount_RNA", "nFeature_RNA", "percent.mt"). Top50 Principal Components (PC) based on the top 5000 variable features were calculated. Based on the PCA elbow plot, the shared Nearest-neighbor graph has been calculating on the top 20 PCs. Finally, Louvain clustering was performed with a resolution of 0.8 and clusters were visualized on Uniform Manifold Approximation and Projection (UMAP). Putative cell types were identified using the sctype R package (11), irrelevant non-CD3$^+$ cell types (e.g. cluster 15, cluster 17, cluster 18 or cluster 20) were considered as contaminants. After applying quality filtering and removing cell-type contamination, a total of 6476 cells out of 15784 have been selected. Non-parametric Wilcoxon test was used to identify marker genes. Adjusted *P*-value below 0.05 was considered as significant. The scRNA-seq data are deposited in the database GEO repository under the GEO accession number GSE242334.

Flow cytometry

Cells were harvested from *in vitro* experiments or isolated from mouse organs and stained for surface markers for 20 minutes at 4°C in staining buffer (PBS with 0.5% BSA and 0.01% sodium azide). All antibodies used in the study are listed in **Suppl. Table 4**. Zombie NIR Fixable Viability Kit (BioLegend, catalog # 423106) was used according to the manufacturer's instructions to exclude dead cells in all analyses. For the evaluation of intracellular antigens, cells were then fixed (BD Cytofix/Cytoperm Fixation/Permeabilization Kit, catalog # 554714) for 45 minutes at 4°C, washed, and stained with antibodies targeting intracellular markers in permeabilization buffer for 30 minutes at 4°C.

For intracellular cytokine detection, fresh single-cell suspensions were incubated in a 96-well, round-bottom plate for 5 hours with the eBioscience Cell Stimulation Cocktail plus protein transport inhibitors (Fisher Scientific, catalog # 00-4975-93). Cells were stained for surface markers, before fixation, permeabilization, and intracellular staining. Multiparametric flow cytometry analyses were performed using a Celesta cytometer (BD Biosciences).

Spectral Flow cytometry

For high dimensional analysis of spectral flow cytometry data, cells were stained using the antibodies specified in **Suppl. Table 3**.

Cells were incubated with along with True Stain FcX (BioLegend) and True Stain Monocyte Blocker (BioLegend) to prevent unspecific binding. For dead cell exclusion, cells were stained with Zombie NIR fixable viability dye (BioLegend, 1:500). Surface antigen staining was performed in PBS for 20 minutes at 4°C. The cells were then washed in PBS and fixed with the Foxp3 / Transcription Factor Staining Buffer Set (ThermoFisher) according to the manufacture's instruction. For intracellular cytokine staining, cells were incubated with the respective intracellular antibodies diluted in Permeabilization buffer (ThermoFisher). After washing, cells were resuspended in PBS and acquired on Cytek Aurora (Cytek Biosciences). Data pre-processing was carried out in FlowJo (TreeStar) for singlets and dead cell exclusion and $CD45^+$ cell selection.

Compensated and pre-gated cells were then imported into R studio using R version 4.0.2 using the read.flowSet() function of the flowCore package. The flow cytometry data was then transformed using a hyperbolic arcsine (arcsinh) transformation and percentile normalized to obtain expression values between 0 and 1. This was followed by unifold manifold approximation and projection (UMAP) using the *umap* package. Automated clustering and metaclustering of the percentile normalized data was performed with the FlowSOM package. This was followed by expert-guided merging of some clusters based on their median marker expression profile.

Differential abundance analysis was performed with a Wilcoxon test, differential expression was calculated using the limma test implemented in the diffCyt package.

Analysis of human scRNA sequencing datasets

To quantify *HAVCR2*, *LGALS9*, and *HMGB1* expression in healthy bone marrow and AML cells at diagnosis, we used provided cell type annotations (12). We scaled data with the Seurat version 4.9.9.9050 function ScaleData and scale.max = 4. Next, we selected the relevant cell types and visualized gene expression using the ComplexHeatmap package version 2.16.0 (13). Student's t-tests between healthy donor and AML patient Tc yielded $P < 2.2 \times 10^{-16}$ for *HAVCR2*, *LGALS9*, and *HMGB1*.

These procedures are described on https://github.com/petervangalen/reanalyze-aml2019/blob/main/230830_Heatmaps_Zeiser.R."

Focused reanalyses of *HAVCR2, CEACAM1, HMGB1, LGALS9* and *PTDSS1* expression across 5 published datasets of AML and healthy donor bone marrow single cell RNA sequencing profiles (12, 14-17) were performed using the available count matrices in R with the Seurat package version 4.3.0 (18). After creation of Seurat objects for each study according to standard best practices, cell types in each dataset were annotated by projection of the bone marrow reference dataset provided by Seurat4 following supervised principal component analysis (sPCA) (10) as described in the respective vignette. Scaled gene expression was visualized using the FeaturePlot() function of Seurat. Relevant code is available under https://github.com/liviuspenter/TIM3_AML.

Survival analysis

Reanalyses of survival differences in AML was based on the expression level of *HAVCR2* across published datasets of AML cohorts (19). RNAseq data was collected from the GenomicDataCommons (GDC) library. A query was used to download public accessible STAR-Counts: TARGET-AML. Additionally, the data were filtered by tissue type: Primary Blood Derived Cancer - Peripheral Blood, Primary Blood Derived Cancer - Bone Marrow, Blood Derived Normal, Bone Marrow Normal, Recurrent Blood Derived Cancer - Peripheral Blood and Recurrent Blood Derived Cancer - Bone Marrow. High *HAVCR2* expression was determined by calculating the mean. Gene expression more than one standard deviation above the mean was considered as high expression.

References

1. Dixon KO, Tabaka M, Schramm MA, Xiao S, Tang R, Dionne D, Anderson AC, Rozenblatt-Rosen O, Regev A, and Kuchroo VK. TIM-3 restrains anti-tumour immunity by regulating inflammasome activation. *Nature.* 2021;595(7865):101-6.
2. Mathew NR, Baumgartner F, Braun L, O'Sullivan D, Thomas S, Waterhouse M, Muller TA, Hanke K, Taromi S, Apostolova P, et al. Sorafenib promotes graft-versus-leukemia activity in mice and humans through IL-15 production in FLT3-ITD-mutant leukemia cells. *Nat Med.* 2018;24(3):282-91.
3. Uhl FM, Chen S, O'Sullivan D, Edwards-Hicks J, Richter G, Haring E, Andrieux G, Halbach S, Apostolova P, Buscher J, et al. Metabolic reprogramming of donor T cells enhances graft-versus-leukemia effects in mice and humans. *Sci Transl Med.* 2020;12(567).
4. Wilhelm K, Ganesan J, Muller T, Durr C, Grimm M, Beilhack A, Krempl CD, Sorichter S, Gerlach UV, Juttner E, et al. Graft-versus-host disease is enhanced by extracellular ATP activating P2X7R. *Nat Med.* 2010;16(12):1434-8.
5. Schwab L, Goroncy L, Palaniyandi S, Gautam S, Triantafyllopoulou A, Mocsai A, Reichardt W, Karlsson FJ, Radhakrishnan SV, Hanke K, et al. Neutrophil granulocytes recruited upon translocation of intestinal bacteria enhance graft-versus-host disease via tissue damage. *Nat Med.* 2014;20(6):648-54.
6. Kaplan DH, Anderson BE, McNiff JM, Jain D, Shlomchik MJ, and Shlomchik WD. Target antigens determine graft-versus-host disease phenotype. *J Immunol.* 2004;173(9):5467-75.
7. Prestipino A, Emhardt AJ, Aumann K, O'Sullivan D, Gorantla SP, Duquesne S, Melchinger W, Braun L, Vuckovic S, Boerries M, et al. Oncogenic JAK2(V617F) causes PD-L1 expression, mediating immune escape in myeloproliferative neoplasms. *Sci Transl Med.* 2018;10(429).
8. Arguello RJ, Combes AJ, Char R, Gigan JP, Baaziz AI, Bousiquot E, Camosseto V, Samad B, Tsui J, Yan P, et al. SCENITH: A Flow Cytometry-Based Method to Functionally Profile Energy Metabolism with Single-Cell Resolution. *Cell Metab.* 2020;32(6):1063-75 e7.
9. Zheng GX, Terry JM, Belgrader P, Ryvkin P, Bent ZW, Wilson R, Ziraldo SB, Wheeler TD, McDermott GP, Zhu J, et al. Massively parallel digital transcriptional profiling of single cells. *Nat Commun.* 2017;8(14049.
10. Hao Y, Hao S, Andersen-Nissen E, Mauck WM, 3rd, Zheng S, Butler A, Lee MJ, Wilk AJ, Darby C, Zager M, et al. Integrated analysis of multimodal single-cell data. *Cell.* 2021;184(13):3573-87 e29.
11. Ianevski A, Giri AK, and Aittokallio T. Fully-automated and ultra-fast cell-type identification using specific marker combinations from single-cell transcriptomic data. *Nat Commun.* 2022;13(1):1246.
12. van Galen P, Hovestadt V, Wadsworth Ii MH, Hughes TK, Griffin GK, Battaglia S, Verga JA, Stephansky J, Pastika TJ, Lombardi Story J, et al. Single-Cell RNA-Seq Reveals AML Hierarchies Relevant to Disease Progression and Immunity. *Cell.* 2019;176(6):1265-81 e24.
13. Gu Z. Complex heatmap visualization. *iMeta Science.* 2022.
14. Abbas HA, Hao D, Tomczak K, Barrodia P, Im JS, Reville PK, Alaniz Z, Wang W, Wang R, Wang F, et al. Single cell T cell landscape and T cell receptor repertoire profiling of AML in context of PD-1 blockade therapy. *Nat Commun.* 2021;12(1):6071.
15. Beneyto-Calabuig S, Merbach AK, Kniffka JA, Antes M, Szu-Tu C, Rohde C, Waclawiczek A, Stelmach P, Grassle S, Pervan P, et al. Clonally resolved single-cell multi-omics identifies routes of cellular differentiation in acute myeloid leukemia. *Cell Stem Cell.* 2023;30(5):706-21 e8.

16. Penter L, Liu Y, Wolff JO, Yang L, Taing L, Jhaveri A, Southard J, Patel M, Cullen NM, Pfaff KL, et al. Mechanisms of response and resistance to combined decitabine and ipilimumab for advanced myeloid disease. *Blood.* 2023;141(15):1817-30.
17. Huo Y, Wu L, Pang A, Li Q, Hong F, Zhu C, Yang Z, Dai W, Zheng Y, Meng Q, et al. Single-cell dissection of human hematopoietic reconstitution after allogeneic hematopoietic stem cell transplantation. *Sci Immunol.* 2023;8(81):eabn6429.
18. Stuart T, Butler A, Hoffman P, Hafemeister C, Papalexi E, Mauck WM, 3rd, Hao Y, Stoeckius M, Smibert P, and Satija R. Comprehensive Integration of Single-Cell Data. *Cell.* 2019;177(7):1888-902 e21.
19. National Cancer Institute: TARGET Data Matrix. *https://targetncinihgov/dataMatrix/*.
